# AID and TET2 cooperate to demethylate *Irf4* for plasma cell fate in germinal center B cells

**DOI:** 10.1084/jem.20260096

**Published:** 2026-04-27

**Authors:** Minghui He, Roberta D’Aulerio, Lia G. Pinho, Evangelos Doukoumopoulos, Tracer Yong, Rhaissa C. Vieira, Mariana M.S. Oliveira, Laura Eiben, Manon Termote, Rômulo G. Galvani, Saikiran Sedimbi, Christina Seitz, Nikolai V. Kuznetsov, Maria A. Zuriaga, Daisuke Kitamura, José J. Fuster, Vinicius Cotta-de-Almeida, Lena Ström, Pia Dosenovic, Søren E. Degn, Lisa S. Westerberg

**Affiliations:** 1 Department of Microbiology Tumor and Cell Biology, Karolinska Institutet, Stockholm, Sweden; 2 National Laboratory of Scientific Computing-LNCC, Labinfo-Bioinformatics Laboratory, Petrópolis, Brazil; 3 Laboratory for Thymus Research (LPT), Oswaldo Cruz Institute, Oswaldo Cruz Foundation (FIOCRUZ), Rio de Janeiro, Brazil; 4 Centro Nacional de Investigaciones Cardiovasculares (CNIC), Madrid, Spain; 5 Research Institute for Biomedical Sciences, Tokyo University of Science, Noda, Japan; 6 Centro de Investigación Biomédica en Red de Enfermedades Cardiovasculares (CIBERCV), Madrid, Spain; 7 Department of Cell and Molecular Biology, Karolinska Institutet, Stockholm, Sweden; 8 Department of Biomedicine, Aarhus University, Aarhus C, Denmark

## Abstract

Activation-induced cytidine deaminase (AID) is essential for B cell affinity maturation. We investigated why AID deficiency gives rise to giant germinal centers (GCs) using the AID^R112H^ mouse model that is devoid of AID activity. The increased GC response was associated with accumulation of GC B cells in the light zone in immunized AID^R112H^ mice. AID^R112H^ GC B cells had reduced capacity to upregulate IRF4 to initiate plasma cell differentiation, leading to accumulation of a transitional GC population with reduced GL7 expression. Genetic introduction of a high-affinity B cell receptor was unable to restore plasma cell differentiation of AID^R112H^ B cells, while ectopic expression of catalytically active AID rescued plasma cell generation. AID and ten-eleven translocation 2 (TET2) synergistically facilitated demethylation of the *Irf4* promoter/enhancer, and this was impeded in AID^R112H^ cells. These data reveal a B cell–intrinsic mechanism that governs the plasma cell fate decision through epigenetic remodeling mediated by AID in cooperation with TET2.

## Teaser

He et al. identify AID as a key initiator of plasma cell differentiation, beyond its established roles in SHM and CSR. They show that AID cooperates with TET2 to initiate plasma cell commitment by promoting demethylation of the Irf4 locus.

## Graphical Abstract

**Figure:**
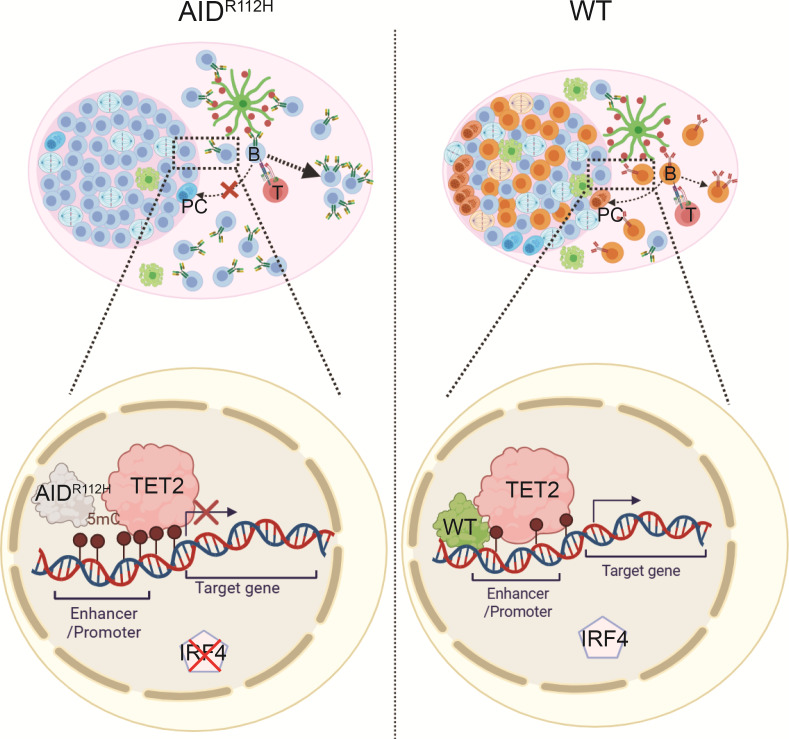

## Introduction

A hallmark of the humoral immune response is the formation of isotype-switched, long-lived plasma cells (PCs) and memory B cells in germinal centers (GCs). A mature GC can be histologically divided into a dark zone (DZ) and a light zone (LZ). Proliferative B cells populating the DZ highly express activation-induced cytidine deaminase (AID) to diversify their B cell receptors (BCRs) through somatic hypermutation (SHM) and Ig class-switch recombination (CSR) (Di Noia and Neuberger, 2007; Stavnezer et al., 2008; Muramatsu et al., 1999; Muramatsu et al., 2000). The somatically mutated B cells test their BCR affinity through capturing antigens deposited on follicular dendritic cells (FDCs) and presentation to T follicular helper (Tfh) cells in the LZ (Victora et al., 2010; Victora and Nussenzweig, 2012). External signals from FDCs and Tfh cells intertwined with epigenetic and transcriptional programs to orchestrate GC B cell differentiation into PCs (Venturutti and Melnick, 2021).

As B cells transit through the GC response to form PCs, the genomic architecture is reconfigured substantially (Papin et al., 2022) due to epigenetic remodeling including alterations in histone modification and DNA methylation (Venturutti and Melnick, 2021). To counteract the passive DNA demethylation induced by rapid cell proliferation, GC B cells express a DNA methyltransferase, DNMT1, to maintain the B cell–specific DNA methylome pattern (Shaknovich et al., 2011). Locus-specific DNA demethylation occurs in GC B cells to ensure normal GC B cell differentiation (Wu et al., 2022), and is mediated by the members of the ten-eleven translocation (TET) protein family, including TET2 and TET3 (Wu and Zhang, 2017). TET proteins can mediate iterative oxidation of 5-methylcytosine (5mC), therefore generating 5-hydroxymethylcytosine (5hmC), 5-formylcytosine (5fC), and 5-carboxylcytosine (5caC) (Pastor et al., 2013). 5hmC, 5fC, and 5caC can lead to passive DNA demethylation through replication-dependent dilution of 5mC. Alternatively, 5fC and 5caC can be excised by thymine DNA glycosylase (TDG) that is coupled with the base excision repair (BER) pathway to mediate active DNA demethylation (Wu and Zhang, 2017). The loci undergoing DNA hypomethylation in GC B cells are overrepresented at B cell enhancers that cover key genes for PC formation, *Irf4*, *Prdm1*, and *Xbp1* (Barwick et al., 2016). Concomitantly, upregulation of IRF4 and downregulation of BCL6 and PAX5 precede the PC differentiation (Nutt et al., 2015). This coincides with a progressive DNA demethylation at the *Irf4* locus from naive B cells to the terminally differentiated PC stage and is mediated by TET proteins (Fujii et al., 2020).

To initiate SHM and CSR, AID is associated with the transcription machinery and specifically targeted to the Ig variable and switch regions, where AID can deaminate cytidine into uracil at the single-stranded DNA (ssDNA) exposed during transcription (Nambu et al., 2003; Pavri et al., 2010; Kodgire et al., 2013; Maul et al., 2014; Wang et al., 2014). The resulting U:G mismatch can be sensed and removed by uracil-DNA glycosylase (UNG), eliciting a DNA damage response (Daniel and Nussenzweig, 2013; Yan et al., 2007; Rada et al., 2004; Methot and Di Noia, 2017). Repair of AID-induced DNA damage will result in SHM in the variable region and CSR in the constant region of the Ig locus (Di Noia and Neuberger, 2007; Stavnezer et al., 2008). AID deficiency leads to type II hyper-IgM syndrome (Revy et al., 2000) with a hotspot mutation in arginine 112-to-histidine mutation (AID^R112H^) that abolishes the catalytic activity of the AID (Kohli et al., 2009; Carpenter et al., 2010; Wang et al., 2010; Prochnow et al., 2007). In addition to the complete absence of SHM and CSR, both type II hyper-IgM patients and AID-deficient mice possess lymphadenopathy caused by giant and persistent GCs (Muramatsu et al., 2000; Revy et al., 2000; Dahlberg et al., 2014). In addition to BCR diversification, it has been suggested that AID mediates active demethylation probably via deamination of 5mCs in various non–B cell models (Morgan et al., 2004; Ramiro and Barreto, 2015).

Using our previously established mouse model for the type II hyper-IgM syndrome (Dahlberg et al., 2014), we found that lymphadenopathy in AID^R112H^ mice was associated with accumulation of GC B cells with low expression of GL7 at a transitional GC (tGC) stage. This was caused by reduced capacity to induce PC differentiation due to compromised upregulation of IRF4 in AID^R112H^ GC B cells. Genetic introduction of a high-affinity BCR failed to rescue the PC differentiation in AID^R112H^ GC B cells; instead, ectopic expression of the wild-type (WT) AID protein fully restored the capacity to generate PCs. Our data reveal that AID and TET2 cooperate at the *Irf4* promoter/enhancer, and that AID deamination activity was required for efficient DNA demethylation of the *Irf4* gene for commitment to the PC lineage.

## Results

### Increased GC response with accumulation of LZ B cells in AID^R112H^ mice

**Figure 1.**
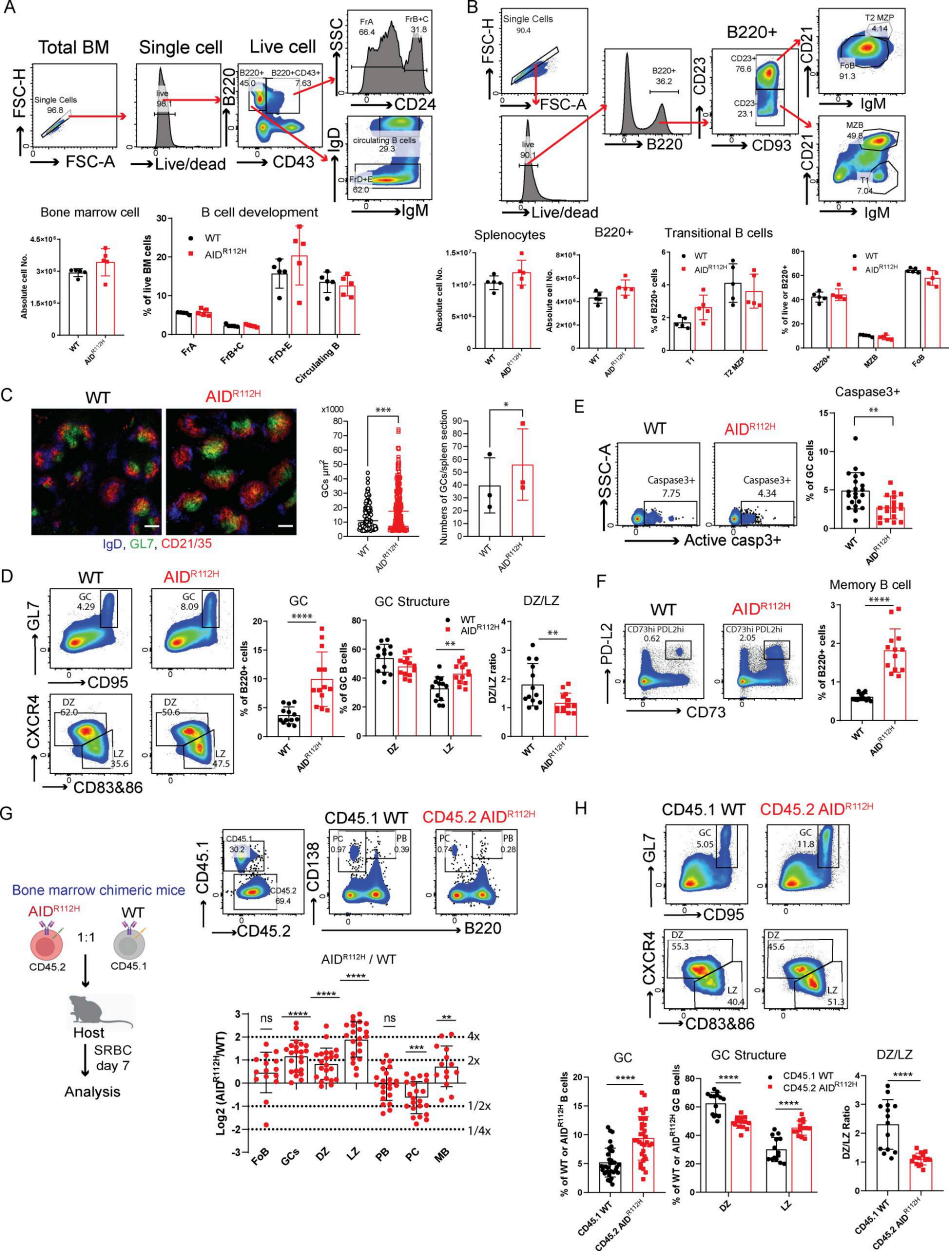
**B cell development and differentiation in AID** ^**R112H**^ **mice and bone marrow chimeric mice. (A)** B cell development in bone marrow by the Hardy classification. Upper panels: Representative FACS plots for gating strategy. Lower panels: Total cell number of bone marrow, P = 0.032, WT versus AID^R112H^, unpaired *t* test (*n* = 5, one experiment); % of FrA (fraction A), FrB + C (fraction B + C), FrD + E (fraction D + E), nonsignificant when comparing WT versus AID^R112H^ in all groups. **(B)** B cell development in the spleen. Upper panels: Representative FACS plots for gating. Lower panels: Quantifications, total spleen cells, nonsignificant; total B220^+^ cells, P = 0.044, unpaired *t* test (*n* = 5, one experiment); % of T1, T2 MZP; B220^+^, MZB, FoB, nonsignificant for all groups, WT versus AID^R112H^, unpaired *t* test (*n* = 5, one experiment). **(C)** GC in spleen section on day 7 upon SRBC immunization. Blue: IgD; green: GL7; red: CD21/35. Scale bar, 200 µm. **(D)** GC response on day 7 upon SRBC immunization. Left panels: representative FACS plot for GC, DZ, and LZ; Right panels: quantification, WT versus AID^R112H^; GC, P < 0.0001; DZ, P = 0.137; LZ, P = 0.0037. DZ/LZ ratio, P = 0.0097, unpaired *t* test (*n* = 13, three experiments). **(E)** Active caspase 3^+^ apoptotic cells in GC. FACS plots and quantification, WT versus AID^R112H^, P < 0.01, *t* test (*n* = 19–21, four experiments). **(F)** Memory B cell differentiation in the spleen. FACS plot and quantification, P < 0.01, unpaired *t* test (*n* = 13, three experiments). **(G)** Antigen-dependent B cell differentiation in the spleen of bone marrow chimeric mice. Upper panel: Experiment scheme. Middle panel: FACS plot of splenic PB and PCs in the chimeric mice. Lower panel: Quantification, y axis: log2 of measured AID^R112H^/WT-to-grafted AID^R112H^/WT ratio; GC, DZ, and LZ: P < 0.0001; PC: P < 0.001; MB: P < 0.01, one-sample *t* test (*n* = 14–22, three experiments). **(H)** GC response in the bone marrow chimeric mice. Left panels: Representative FACS plot; right: quantification, CD45.1 WT versus CD45.2 AID^R112H^; GC, P < 0.0001, paired *t* test (*n* = 33, four experiments); DZ, P < 0.0001; LZ, P < 0.0001, two-way ANOVA (*n* = 14, three experiments); DZ/LZ ratio, P < 0.0001, paired *t* test (*n* = 14, three experiments). Value and error bar: mean ± SD. MB, memory B cell; T1, transitional 1; T2 MZP, transitional 2 marginal zone progenitor; MZB, marginal zone B cells; FoB, follicular B cells. *P < 0.05, **P < 0.01, ***P < 0.001.

**Figure S1.**
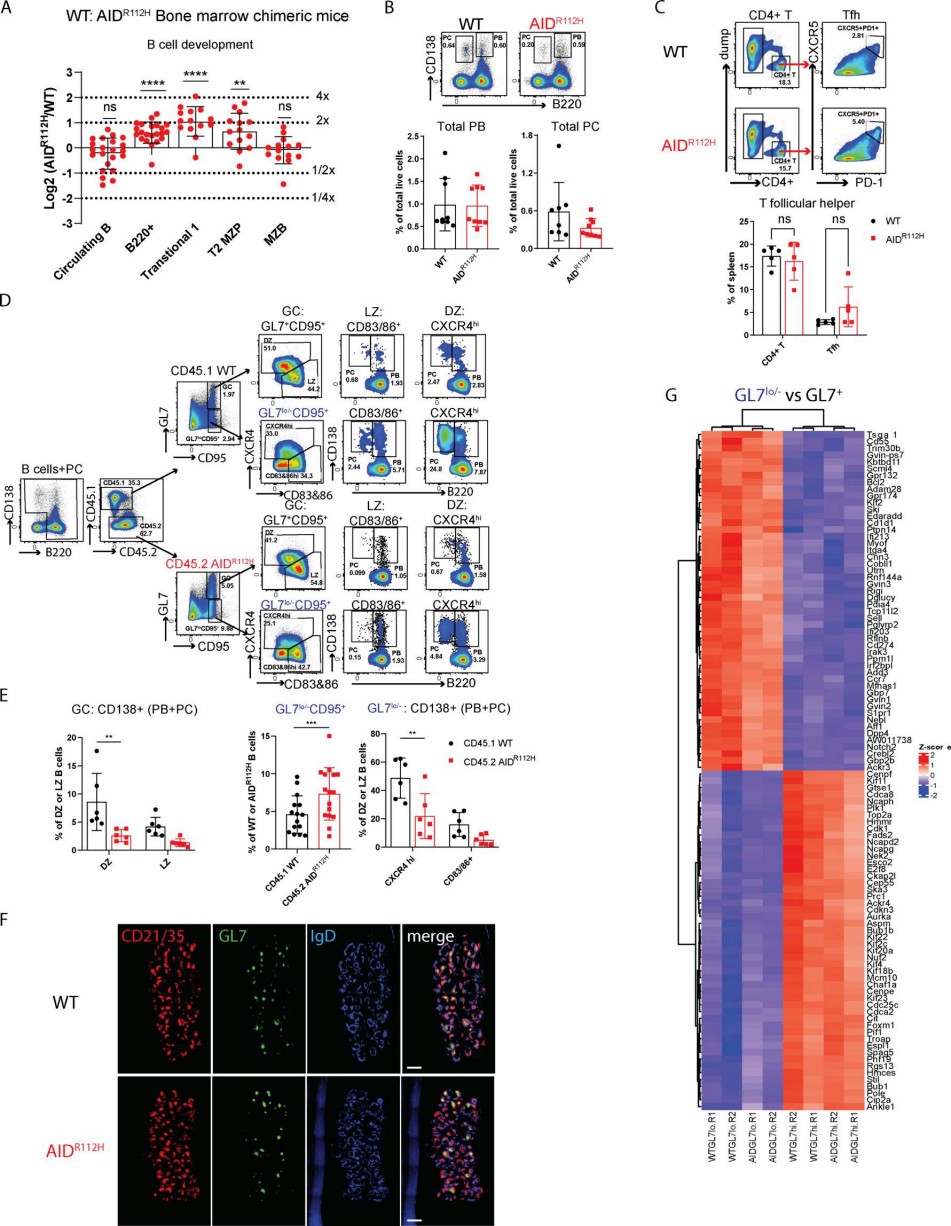
**B cell development and differentiation in bone marrow chimeric mice. (A)** B cell development in the bone marrow chimeric mice. y axis: log2 of measured AID^R112H^/WT-to-grafted AID^R112H^/WT ratio, value 1 = twofold advantage, −1 = twofold disadvantage of AID^R112H^; B220^+^, transitional 1: P < 0.0001; T2 MZP, P < 0.01. one-sample *t* test (*n* = 14–22, three experiments). **(B)** PC differentiation in the spleen. FACS plot and quantification: PB and PC, P > 0.05, unpaired *t* test (*n* = 9, two experiments). **(C)** Tfh cells in the spleen. FACS plot and quantification, nonsignificant. ANOVA (*n* = 5, two experiments). **(D)** Gating strategy to identify PB and PC in GC and GL7^lo/−^CD95^+^ cell population from CD45.1 WT cells and CD45.2 AID^R112H^ cells. PB: B220^+^CD138^+^, PC: B220^−^CD138^+^, representative FACS plots. **(E)** Quantification for D, CD138^+^ cells (PB + PC) in DZ and LZ. DZ: P = 0.0024; LZ: nonsignificant, two-way ANOVA (*n* = 6, two experiments); GL7^lo/−^CD95^+^ cells in CD45.1 WT cells and CD45.2 AID^R112H^ cells, P < 0.001, paired *t* test (*n* = 15, three experiments); CD138^+^ cells in different subsets from GL7^lo/−^CD95^+^ cell population: CXCR4^hi^: P = 0.0014; CD83/86^+^: P = 0.225, two-way ANOVA (*n* = 6, two experiments). **(F)** Immunohistochemistry of GC from full spleen sections of WT and AID^R112H^ mouse 7 days after SRBC immunization. Red: CD21/35; green: GL7; blue: IgD. Scale bar, 500 µm. **(G)** RNA-sequencing heatmap for the top 50 DEGs comparing GL7^lo/−^CD95^+^ versus GL7^+^CD95^+^. T2 MZP, transitional 2 marginal zone progenitor; DEGs, differentially expressed genes. *P < 0.05, **P < 0.01, ***P < 0.001.

The expression of AID is detectable throughout B cell development with the highest expression in GC DZ B cells (Kuraoka et al., 2011; Meyers et al., 2011). B cell development in bone marrow and spleen was comparable between AID^R112H^ and WT mice (Fig. 1, A and B). To examine the B cell–intrinsic effect of the AID^R112H^ mutation and to exclude an effect of the microenvironment in AID^R112H^ mice, we generated mixed bone marrow chimeric mice that were reconstituted with a 1:1 ratio of CD45.1 WT and CD45.2 AID^R112H^ cells. Circulating B cells, follicular B cells, and marginal zone B cells were comparable in WT and AID^R112H^ mice (Fig. S1 A), suggesting that the antigen-independent phase of B cell development in the bone marrow and spleen was not impaired by the AID^R112H^ mutation.

Immunohistochemistry and flow cytometry analysis revealed that AID^R112H^ mice had increased GC response with elevated GC B cells when compared to WT mice (Fig. 1, C and D) (Dahlberg et al., 2014). AID^R112H^ GC B cells had a reduced active caspase 3^+^ population (Fig. 1 E), which is consistent with previous findings of reduced apoptosis in AID knockout GC B cells (Zaheen et al., 2009; Mayer et al., 2017). To study the GC response, we analyzed sheep red blood cell (SRBC)–immunized GC B cells (GL7^+^CD95^+^) and, among these LZ (CXCR4^lo^CD83/86^hi^) and DZ (CXCR4^hi^CD83/86^lo^) populations, the generation of spleen plasmablasts (PBs, B220^+^CD138^+^), PCs (B220^−^CD138^+^), and memory B cells (PD-L2^+^CD73^+^). The GC in AID^R112H^ mice had an altered structure, with increased LZ population leading to a reduced DZ-to-LZ ratio in AID^R112H^ mice (Fig. 1 D). AID^R112H^ mice showed a more than twofold increase of the memory B cell population (Fig. 1 F). The output of total spleen PBs and PCs was comparable between AID^R112H^ and WT mice (Fig. S1 B). The proportion of Tfh cells was comparable between AID^R112H^ and WT mice (Fig. S1 C). In the competitive setting of bone marrow chimeric mice, CD45.2 AID^R112H^ B cells had twofold accumulation of GC B cells when compared to CD45.1 WT B cells (Fig. 1, G and H). Moreover, CD45.2 AID^R112H^ GC B cells predominantly located in the LZ, whereas the CD45.1 WT GC B cells were distributed normally between the two zones (Fig. 1 H). This demonstrated a cell-intrinsic effect of AID^R112H^ GC B cells even in an environment where the WT cells could sustain a GC response and where IgG from WT B cells could mediate antibody feedback regulation (Zhang et al., 2013). Consistent with the increased memory B cell population in individually immunized AID^R112H^ mice (Fig. 1 F), CD45.2 AID^R112H^ B cells showed an advantage in memory B cell formation in competition with CD45.1 WT B cells (Fig. 1 G). WT and AID^R112H^ cells contributed equally to differentiation of PBs, and there was a disadvantage of PC formation from the AID^R112H^ GC B cells (Fig. 1 G). These data show that AID deficiency led to aberrant accumulation of GC B cells with preferential localization in the LZ of the GC.

### Unaltered cell proliferation rate but compromised G1/S transition of AID^R112H^ GC B cells

**Figure 2.**
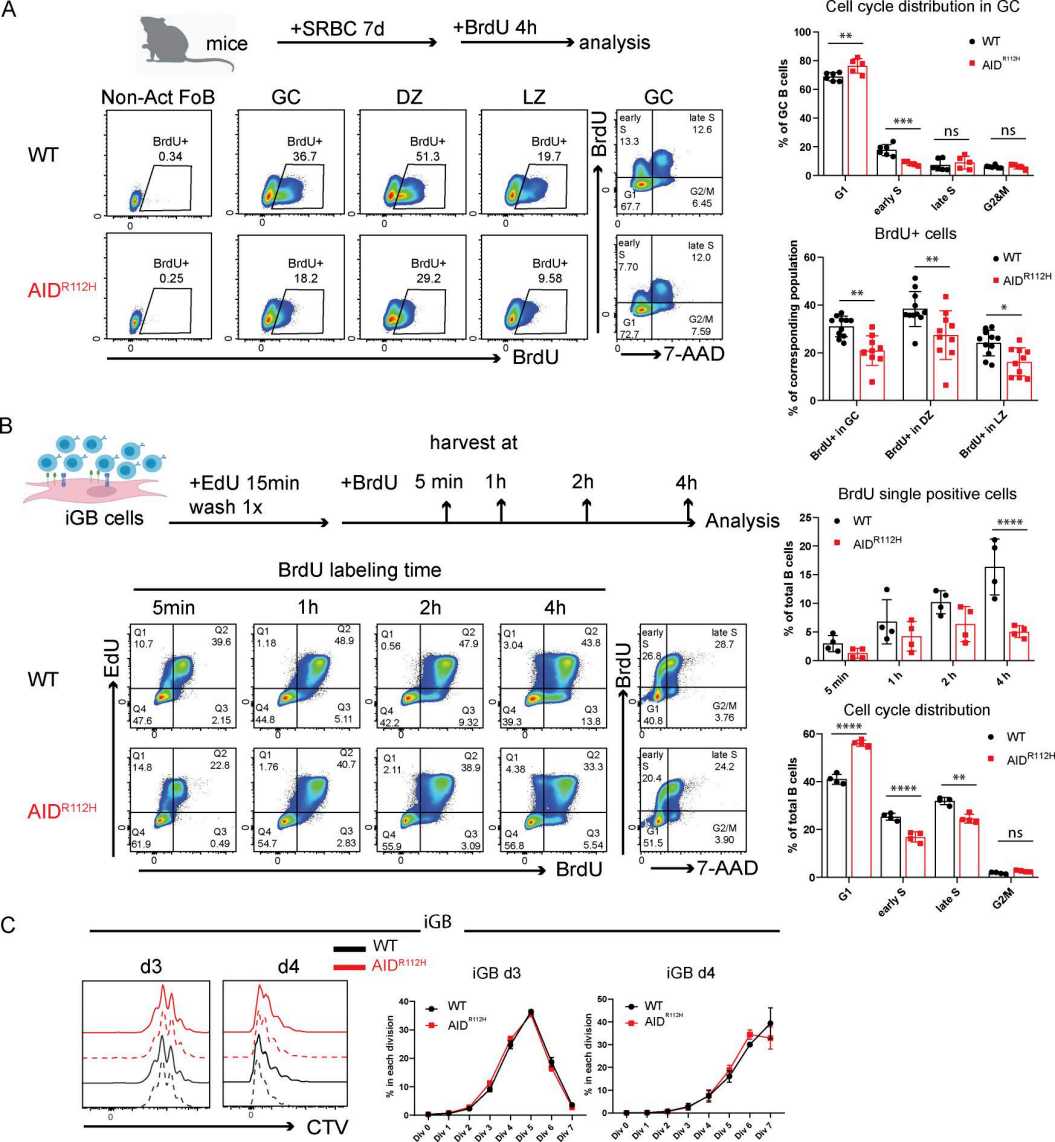
**Reduced G1/S transition of AID** ^**R112H**^ **iGB cells and GC B cells. (A)** In vivo BrdU labeling of GC B cells on day 7 upon SRBC immunization. Schematics of experimental setup and representative FACS plots. Quantification: BrdU^+^ cells in GC: P = 0.0035; DZ: P = 0.0014; LZ: P = 0.0294, two-way ANOVA (*n* = 10–11, two experiments). Cell cycle distribution, G1: P = 0.0027; early S: P = 0.0002; two-way ANOVA (*n* = 5–6, one experiment). 7-AAD on a linear scale to reflect the amount of the DNA content. **(B)** EdU and BrdU double labeling in iGB cells. Schematics for experimental setup and representative FACS plots and quantification, BrdU single-positive cells at 4 h: ****P < 0.0001. Cell cycle distribution: G1, ****P < 0.0001; early S, ****P < 0.0001; late S, **P < 0.01; 7-AAD on a linear scale to reflect the amount of the DNA content (*n* = 4, two experiments). **(C)** iGB cell proliferation rate measured by dilution of CTV. Left: Histogram overlay of WT and AID^R112H^ cells on day 3 and day 4. Right: Quantification of the percentage of cells in each division. Value and error bar: mean ± SD. MFI, mean fluorescence intensity.

To study whether the accumulation of GC B cells in AID^R112H^ mice was caused by increased cell proliferation, SRBC-immunized WT and AID^R112H^ mice were given a 4-h BrdU pulse before analysis on day 7. DZ B cells contained a higher proportion of BrdU^+^ cells when compared to the LZ cells (Fig. 2 A). Surprisingly, GC B cells from AID^R112H^ mice had a decreased BrdU^+^ cell population when compared to GC B cells from WT mice regardless of their location in DZ or LZ (Fig. 2 A). Cell cycle distribution of GC B cells showed an increased G1 population and a decreased early S-phase cell population in AID^R112H^ mice when compared to WT mice (Fig. 2 A). These data suggested a reduced fraction of proliferating B cells in AID^R112H^ GCs compared with WT GCs, which is likely due to compromised G1/S transition of AID^R112H^ GC B cells. This may be caused by an impaired BCR affinity maturation in AID^R112H^ mice, since capacity of GC B cell division is proportional to the amount of captured antigens (Gitlin et al., 2014). To investigate whether there is defective DNA replication/initiation in AID^R112H^ B cells, we set up a system using induced GC B (iGB) cells to separate cells that newly initiated the G1/S transition from those already in the DNA synthesis phase of the cell cycle (Nojima et al., 2011). iGB cells were first given a 15-min EdU pulse, followed by BrdU labeling for 5 min, and 1, 2, and 4 h. Cells that were in the S phase were EdU^+^BrdU^+^, while cells that were in the G1 phase during EdU labeling and had newly entered the S phase during BrdU labeling were EdU^−^BrdU^+^. By measuring the increase of the BrdU single-positive cells, we could measure the rate of G1/S transition. AID^R112H^ iGB cells showed a reduced G1/S transition rate when compared to WT iGB cells (Fig. 2 B). Consistently, cell cycle distribution of iGB cells showed increased G1 and decreased S-phase cell populations among AID^R112H^ iGB cells when compared to WT iGB cells (Fig. 2 B). Despite decreased G1/S transition of AID^R112H^ cells, the global cell proliferation rate in AID^R112H^ iGB cells was unaffected when compared to WT iGB cells as shown by the dilution of cell division dye CellTrace Violet (CTV) (Fig. 2 C).

Together, these data excluded that the lymphadenopathy and accumulation of GC B cells in AID^R112H^ mice were caused by a faster GC B cell proliferation rate.

### AID^R112H^ mice have reduced PC differentiation in GC and accumulate GL7^lo/−^ tGC B cells

**Figure 3.**
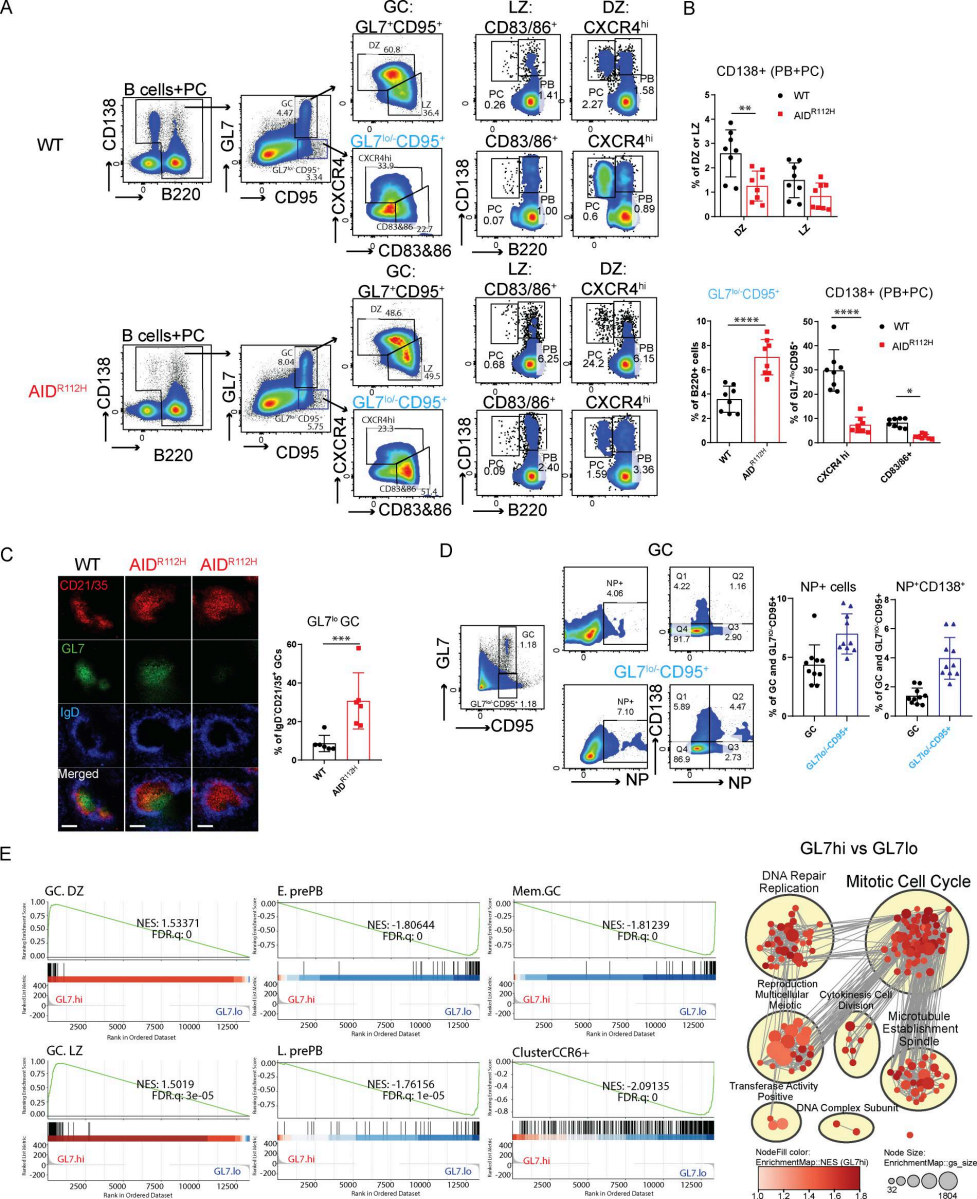
**AID** ^**R112H**^ **mice have reduced** **PC** **differentiation in GCs and accumulate GL7** ^**lo/−**^ **tGC B cells. (A)** Gating strategy of PB and PC in GC and GL7^lo/−^CD95^+^ cell population. PB: B220^+^CD138^+^; PC: B220^−^CD138^+^. FACS plot for GC and GL7^lo/−^CD95^+^ gating used in Fig. S2 E to display other surface markers stained on the same samples. **(B)** Quantifications for A, WT versus AID^R112H^; CD138^+^ cells in DZ, **P = 0.0019; CD138^+^ cells in LZ, nonsignificant. GL7^lo/−^CD95^+^ cells: ****P < 0.0001; CD138^+^ in CXCR4^hi^: ****P < 0.0001; CD138^+^ in CD83/86^+^: P = 0.044, unpaired *t* test (*n* = 8, two experiments). **(C)** Immunohistochemistry of spleen GCs. Red, CD21/35; green, GL7; blue, IgD. Quantification of IgD^−^GL7^−^CD21/35^+^ GCs among total IgD^−^CD21/35^+^ GCs, P = 0.0051, unpaired *t* test (*n* = 6, two experiments). Scale bar, 80 µm. **(D)** NP-specific response of GC and GL7^lo/−^CD95^+^ B cells in WT mice. FACS plots and quantification, GC versus GL7^lo/−^CD95^+^. NP^+^: P = 0.0028; NP^+^CD138^+^: P = 0.0002, unpaired *t* test (*n* = 10, two experiments). **(E)** RNA sequencing comparing GL7^hi^ versus GL7^lo^ GC B cells. Left panel: GSEA using gene signature defined in work (Gómez-Escolar et al., 2022; Glaros et al., 2021); right panel: GSEA network analysis showing gene sets positively enriched in GL7.hi versus GL7.lo. Value and error bar: mean ± SD.

Our data from bone marrow chimeric mice showed an advantage of AID^R112H^ B cells to form GC B cells but a disadvantage to contribute to the PC pool. We reasoned that the large GCs formed in AID^R112H^ mice were caused by a blockage of PC differentiation from GC B cells. PCs can differentiate from both the GC pathway and the extrafollicular pathway (Nutt et al., 2015). To investigate whether there was defective PC formation from GC B cells in AID^R112H^ mice, we performed a new gating strategy as shown in Fig. 3 A to examine the PC differentiation within the GC. Newly formed PBs and PCs were analyzed in the DZ and LZ of the GL7^+^CD95^+^ GC population (Fig. 3 A). AID^R112H^ mice had a reduced CD138^+^ population (including both PBs and PCs) from both DZ and LZ cells when compared to WT mice (Fig. 3 B). The majority of CD138^+^ cells in the LZ were PBs (Fig. 3 A). In contrast, in the DZ population most of the CD138^+^ cells had downregulated the cell surface receptor B220 and become PCs (Fig. 3 A). Similar to individual mice, analysis of bone marrow chimeric mice showed that GC B cells from CD45.2 AID^R112H^ B cells had a reduced CD138^+^ (PB + PC) population when compared to CD45.1 WT GC B cells (Fig. S1, D and E).

In addition to the increased GC B cell population (GL7^+^CD95^+^), AID^R112H^ mice had an increased population of GL7^lo/−^CD95^+^ B cells (Fig. 3 A). Immunohistochemistry of spleen sections confirmed a significant increase of an aberrant type of GCs in AID^R112H^ mice as marked by IgD^−^CD21/35^+^ area with a lower or undetectable level of GL7 compared with WT mice (Fig. 3 C and Fig. S1 F). Based on the surface expression of CXCR4 and CD83/CD86, these cells could be divided into three populations (Fig. 3 A; CXCR4^hi^, CD83/86^hi^, and CXCR4^lo^CD83/86^lo^). To examine whether this population contained a tGC B population destined to leave the GC, we analyzed PC differentiation within the GL7^lo/−^CD95^+^ B cell population. The GL7^lo/−^CD95^+^CXCR4^hi^ B cells contained a higher percentage of CD138^+^ (PB + PC) cells (Fig. 3 B) when compared to DZ B cells in WT mice. This suggested that the GL7^lo/−^CD95^+^ B cells included B cells at a tGC stage, and we refer to them as GL7^lo/−^CD95^+^ tGC B cells. In AID^R112H^ mice, the CD138^+^ (PB + PC) cells among the GL7^lo/−^CD95^+^ B cells were reduced more than threefold when compared to WT mice (Fig. 3 B). To further validate the differentiation stage of the GL7^lo/−^CD95^+^ B cell population and to be able to identify antigen-specific B cells, we immunized WT mice with NP-KLH and examined the GL7^lo/−^CD95^+^ B cells. There were an increased proportion of NP-PE^+^ B cells in the GL7^lo/−^CD95^+^ B population when compared to total GC B cells (Fig. 3 D). Importantly, there was a more than twofold increase of NP^+^CD138^+^ cells in the GL7^lo/−^CD95^+^ B cell population when compared to total GC B cells (Fig. 3 D). In the bone marrow chimeric mice, GL7^lo/−^CD95^+^ CD45.2 AID^R112H^ B cells were increased, while generation of CD138^+^ cells in the GL7^lo/−^CD95^+^ B cells was greatly compromised when compared to CD45.1 WT B cells (Fig. S1, D and E). Altogether, these results suggest that the GL7^lo/−^CD95^+^ cells contained a tGC population and were enriched for CD138^+^ PBs and PCs. Moreover, PC differentiation from the GC response was significantly compromised in the AID^R112H^ mice and characterized by accumulation of the tGC B cells in the GL7^lo/−^CD95^+^ population with a GC LZ phenotype.

**Figure S2.**
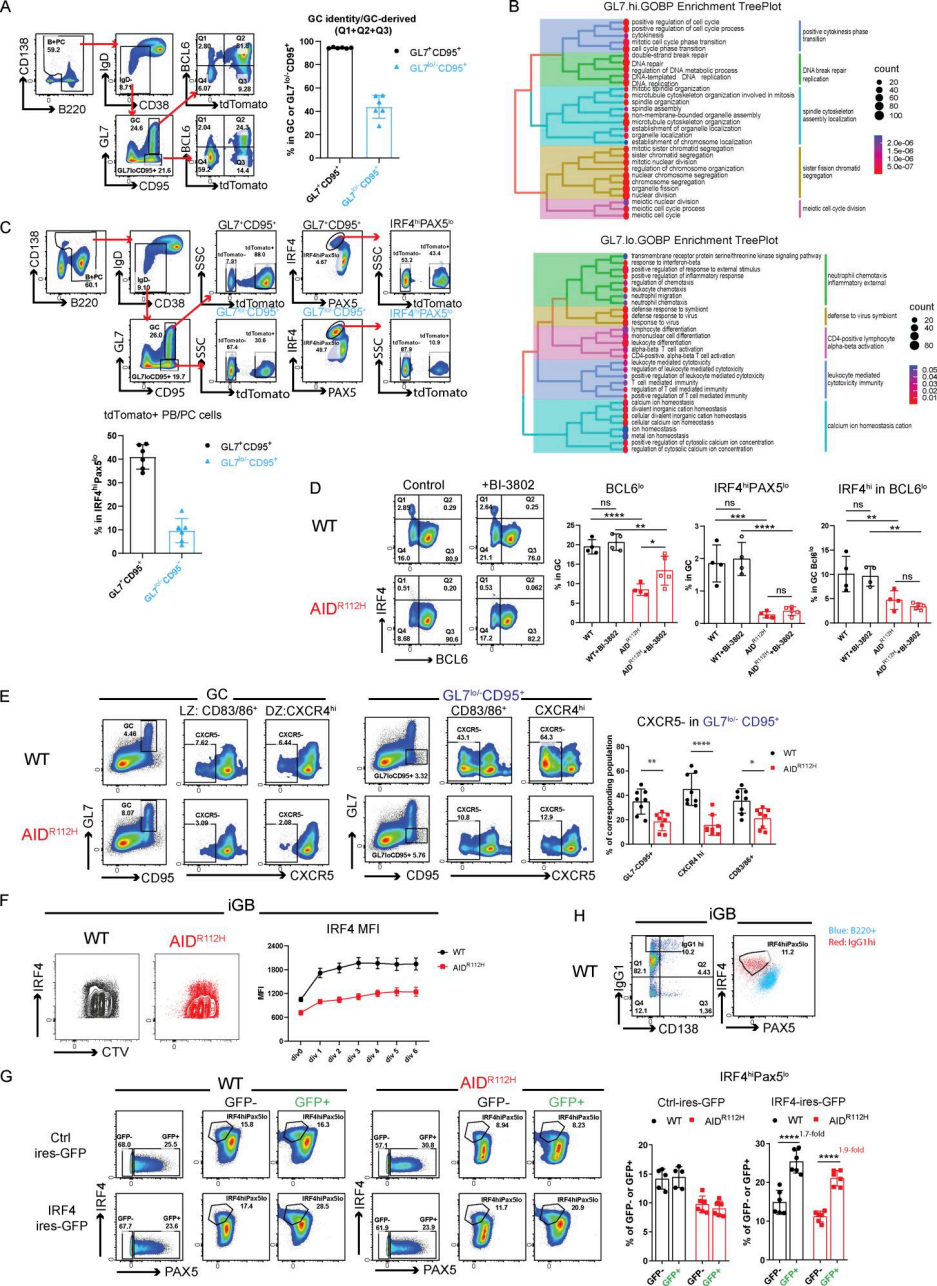
**Fate mapping of GC-experienced B cells in WT mice and impaired IRF4 upregulation and CXCR5 downregulation of GC B cells in AID** ^**R112H**^ **mice. (A)** Proportion of GC-derived B cells in GL7^+^CD95^+^ and GL7^lo/−^CD95^+^ cell populations in S1PR2^ERT2Cre^-RS-loxStoplox-tdTomato mouse model. Gating strategy and quantification of cells with GC identity or GC-derived (BCL6^+^ and tdTomato^+^) (*n* = 6, one experiment). **(B)** GO-term enrichment treeplot for GL7^+^CD95^+^(GL7.hi.) cells and GL7^lo/−^CD95^+^(GL7.lo.) cells. **(C)** GC-derived IRF4^hi^PAX5^lo^ cells in GL7^+^CD95^+^ and GL7^lo/−^CD95^+^ cell populations in S1PR2^ERT2Cre^-RS-loxStoplox-tdTomato mouse model. Representative FACS plots and gating strategy. Quantification of tdTomato+ cells among the IRF4^hi^PAX5^lo^ cells (*n* = 6, one experiment). **(D)** PC differentiation in vivo during the GC response upon BI-3802 treatment for 24 h. Left: Representative FACS plots. Right: Quantification of BCL6^lo^: WT versus AID^R112H^, P < 0.0001; WT + BI-3802 versus AID^R112H^ + BI-3802: P < 0.01; AID^R112H^ versus AID^R112H^ + BI-3802: P < 0.01; IRF4^hi^PAX5^lo^ cells: WT versus AID^R112H^, P < 0.001; WT + BI-3802 versus AID^R112H^ + BI-3802: P < 0.0001; AID^R112H^ versus AID^R112H^ + BI-3802: nonsignificant; IRF4^hi^ within the BCL6^lo^ population: WT versus AID^R112H^, P < 0.01; WT + BI-3802 versus AID^R112H^ + BI-3802: P < 0.01; nonsignificant difference in treated versus nontreated, two-way ANOVA (*n* = 4–5, one experiment). **(E)** Impaired CXCR5 downregulation in AID^R112H^ GL7^lo^CD95^+^ cells. FACS plots and quantification. Same FACS plot on column 1 and column 4 in each row is displayed to show gating strategy for GC and GL7^lo^CD95^+^ population, respectively. FACS plot for GC and GL7^lo/−^CD95^+^ gating used here from the same samples is shown in Fig. 3 A. GL7^lo^CD95^+^: P < 0.01; CXCR4^hi^: P < 0.0001; CD83/86+: P < 0.05, one-way ANOVA (*n* = 8, two experiments). **(F)** Upregulation of IRF4 versus cell division in iGB cells. Representative FACS plot and quantification, IRF4 MFI versus cell division, data from two experiments. **(G)** Overexpression of IRF4 in iGB cells by retroviral transfection. Left: Representative FACS plots. Right: Quantification of IRF4^hi^PAX5^lo^ cells in control and IRF4-transfected cultures. Ctrl-IRES-GFP: no significant changes between WT GFP^−^ versus WT GFP^+^; no significant changes between AID^R112H^ GFP^−^ versus AID^R112H^ GFP^+^; IRF4-IRES-GFP: P < 0.0001, WT GFP^−^ versus WT GFP^+^; P < 0.0001, AID^R112H^ GFP− versus AID^R112H^ GFP^+^, two-way ANOVA (*n* = 5–6, two experiments). **(H)** Increased Ig production of IRF4^hi^PAX5^lo^ cells in WT iGB cultured B cells. Representative FACS plot, IgG1^hi^ cells overlaid with total B220^+^ iGB cells. MFI, mean fluorescence intensity. *P < 0.05, **P < 0.01, ***P < 0.001, ****P < 0.0001.

To define the transcriptome profile of these B cells, we sorted the GL7^lo/−^CD95^+^ cell population (GL7.lo) and compared with sorted GL7^+^CD95^+^ GC cells (GL7.hi) by RNA sequencing. The GL7^lo/−^CD95^+^ B cells clustered together irrespective of the genotype using an unsupervised hierarchical clustering (Fig. S1 G). Gene set enrichment analysis (GSEA) revealed a significant enrichment for signatures of GC DZ and LZ cells in the GL7^hi^ population. In contrast, the GL7^lo/−^CD95^+^ B cell population was significantly enriched for gene signatures of early and late pre-PBs (E.prePB and L.prePB), as well as GC memory B cells (Fig. 3 E) (Gómez-Escolar et al., 2022). Interestingly, GL7^lo/−^CD95^+^ B cells were enriched for a gene signature of CCR6^+^-activated precursor B cells that are a heterogeneous population destined to various cell fates preceding the GC response at days 3–4 upon immunization (Fig. 3 E) (Glaros et al., 2021). Analysis of the GL7^lo/−^CD95^+^ B cell using a GC lineage tracing model (S1PR2-ERT2Cre-RS-loxStoplox-tdTomato [Shinnakasu et al., 2016]) showed that ≥40% of cells in this gate had GC identity (BCL6^+^) and/or were GC-derived (S1PR2-tdTomato^+^, Fig. S2 A). This suggested that the GL7^lo/−^CD95^+^ B cell was a heterogeneous population consisting of both tGC B cells and cells of the extrafollicular pathways. Additionally, the GSEA network analysis revealed a significant enrichment of genes related to cell cycle and DNA repair/replication in the GL7^+^CD95^+^ (GL7.hi) B cell population, while these gene sets were diminished in the GL7^lo/−^CD95^+^ B cell population (Fig. 3 E and Fig. S2 B). This suggested a downregulation of mitotic signatures in the GL7^lo/−^CD95^+^ B cell population. Instead, the GL7^lo/−^CD95^+^ B cells had enriched signatures for cell activation and migration, suggesting an ongoing selection and differentiation process occurring in these cells (Fig. S2 B).

Together, the AID^R112H^ GC B cells differentiated into a tGC phenotype that gradually became GL7^lo/−^ when residing in GCs. These B cells were halted at the pre-PB stage and failed to differentiate into PCs, likely contributing to the increased LZ and GL7^lo/−^CD95^+^ cell population in AID^R112H^ mice.

### AID^R112H^ GC B cells have reduced capacity to upregulate IRF4

**Figure 4.**
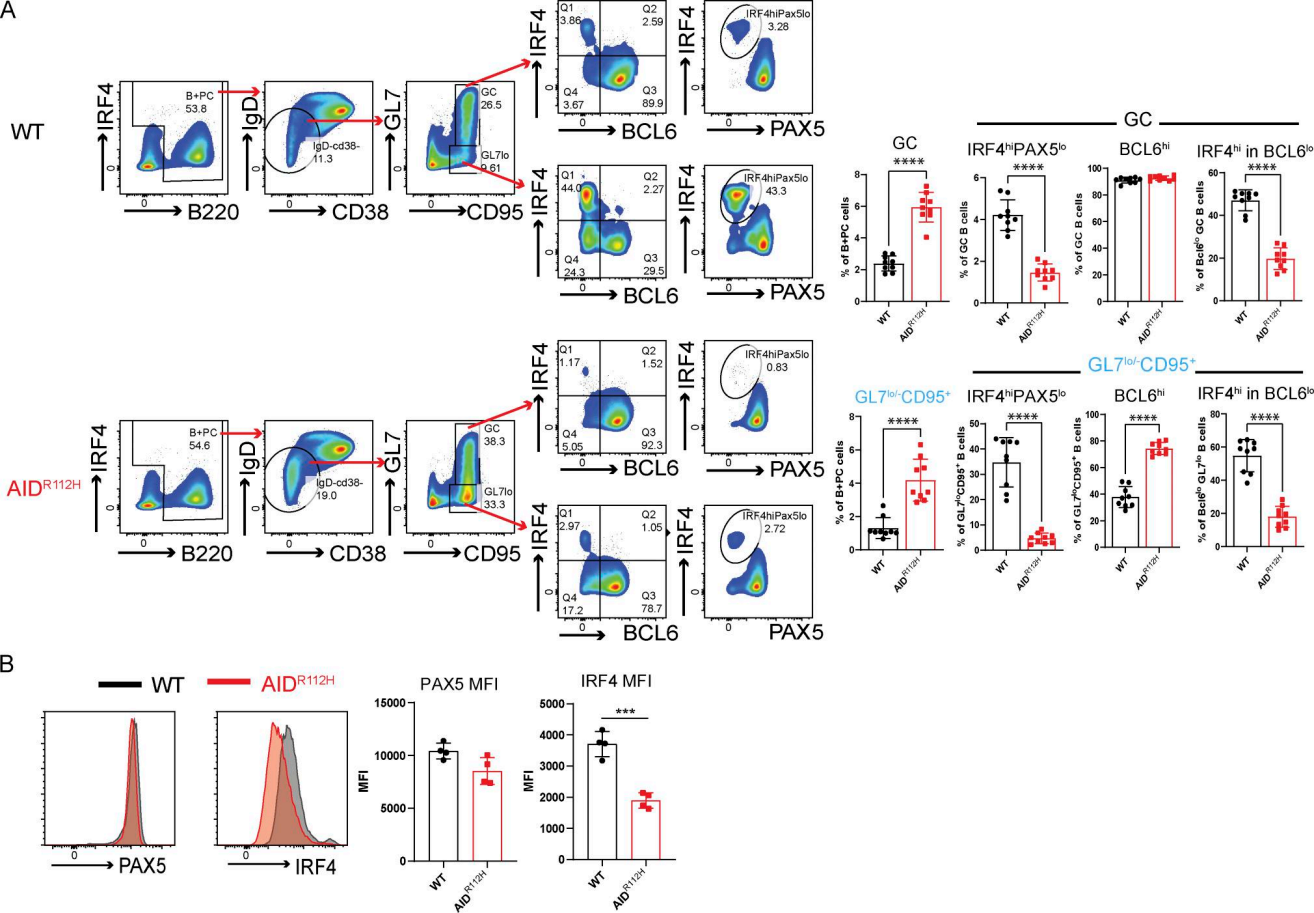
**AID** ^**R112H**^ **GC B cells have reduced capacity to upregulate IRF4. (A)** Gating strategy for expression of transcription factors BCL6, IRF4, and PAX5 in GC and GL7^lo^CD95^+^ B cells. FACS plots and quantification, WT versus AID^R112H^, GC: P < 0.0001; GL7^lo/−^CD95^+^, P < 0.0001; IRF4^hi^PAX5^lo^ in GC: P < 0.0001; IRF4^hi^PAX5^lo^ in GL7^lo/−^CD95^+^, P < 0.0001; IRF4^hi^ in BCL6^lo^ (GC): P < 0.0001; BCL6^hi^ in GL7^lo^CD95^+^: P < 0.0001; IRF4^hi^ in BCL6^lo^ (GL7^lo^CD95^+^): P < 0.0001, unpaired *t* test (*n* = 9–10, three experiments). ***P < 0.001, ****P < 0.0001. **(B)** Expression level of IRF4 and PAX5 in GC B cells. Overlay of histogram of IRF4 and PAX5 acquired by flow cytometry and quantification, WT versus AID^R112H^, IRF4 MFI: P = 0.0006, unpaired *t* test (*n* = 4, two experiments). Nonsignificant differences for PAX5 MFI. Value and error bar: mean ± SD. Value and error bar: mean ± SD.

To investigate the molecular mechanism underlying the defective PC differentiation of AID^R112H^ B cells, we examined the expression of transcription factors BCL6, IRF4, and PAX5 in GC B cells from SRBC-immunized mice. Around 90% of the GL7^+^ GC B cells were BCL6^+^ in both WT and AID^R112H^ mice (Fig. 4 A). The BCL6^**+**^ cells among the GL7^lo^CD95^+^ population were twofold higher in AID^R112H^ mice compared with WT mice (Fig. 4 A), supporting that the BCL6^+^ cells accumulated in the GL7^lo^CD95^+^ population in AID^R112H^ mice. AID^R112H^ GCs showed more than threefold reduction of the IRF4^hi^PAX5^lo^ population when compared to WT GCs (Fig. 4 A). Consistently, the IRF4^hi^PAX5^lo^ population among the GL7^lo/−^CD95^+^ B cells showed more than sevenfold reduction in AID^R112H^ mice when compared to WT mice (Fig. 4 A). This was confirmed using the GC lineage tracing (S1PR2-ERT2Cre-RS-loxStoplox-tdTomato) in WT mice where the GL7^lo/−^CD95^+^ B cells contained IRF4^hi^PAX5^lo^ cells of GC identity (S1PR2-tdTomato^+^, Fig. S2 C). In addition, we found that there was a reduction of the IRF4 protein in AID^R112H^ GC B cells when compared to WT cells, while PAX5 protein expression was comparable between AID^R112H^ and WT GC B cells (Fig. 4 B).

Downregulation of BCL6 precedes PC differentiation (Tunyaplin et al., 2004). Although GC B cells had a comparable level of BCL6^lo^ cells between AID^R112H^ and WT mice, GL7^lo/−^CD95^+^ B cells from AID^R112H^ mice had fewer BCL6^lo^ cells (Fig. 4 A). Moreover, we found that IRF4^hi^ cells only existed in the BCL6^lo^ population. Quantification of the IRF4^hi^ population among the BCL6^lo^ cells showed a significant reduction in AID^R112H^ GCs when compared to WT GCs (Fig. 4 A). By treating the SRBC-immunized mice with BI-3802, a small molecule inhibitor toward BCL6, we detected an increased BCL6^lo^ population in AID^R112H^ GC B cells. However, increased BCL6^lo^ cells in AID^R112H^ GCs did not lead to increased IRF4^hi^PAX5^lo^ cells (Fig. S2 D). This suggests that downregulation of BCL6 was not sufficient for IRF4 upregulation and PC differentiation in AID^R112H^ GC B cells. Moreover, AID^R112H^ B cells had reduced capacity to downregulate CXCR5 on GCs and tGC B cells, providing an explanation for the failure of AID^R112H^ B cells to egress from the GC (Fig. S2 E).

These results suggest that AID^R112H^ B cells downregulated BCL6, but showed reduced capacity to upregulate IRF4 that is required for GC B cells to differentiate into PCs.

### Reduced AID^R112H^ PC formation from in vitro iGB cells

**Figure 5.**
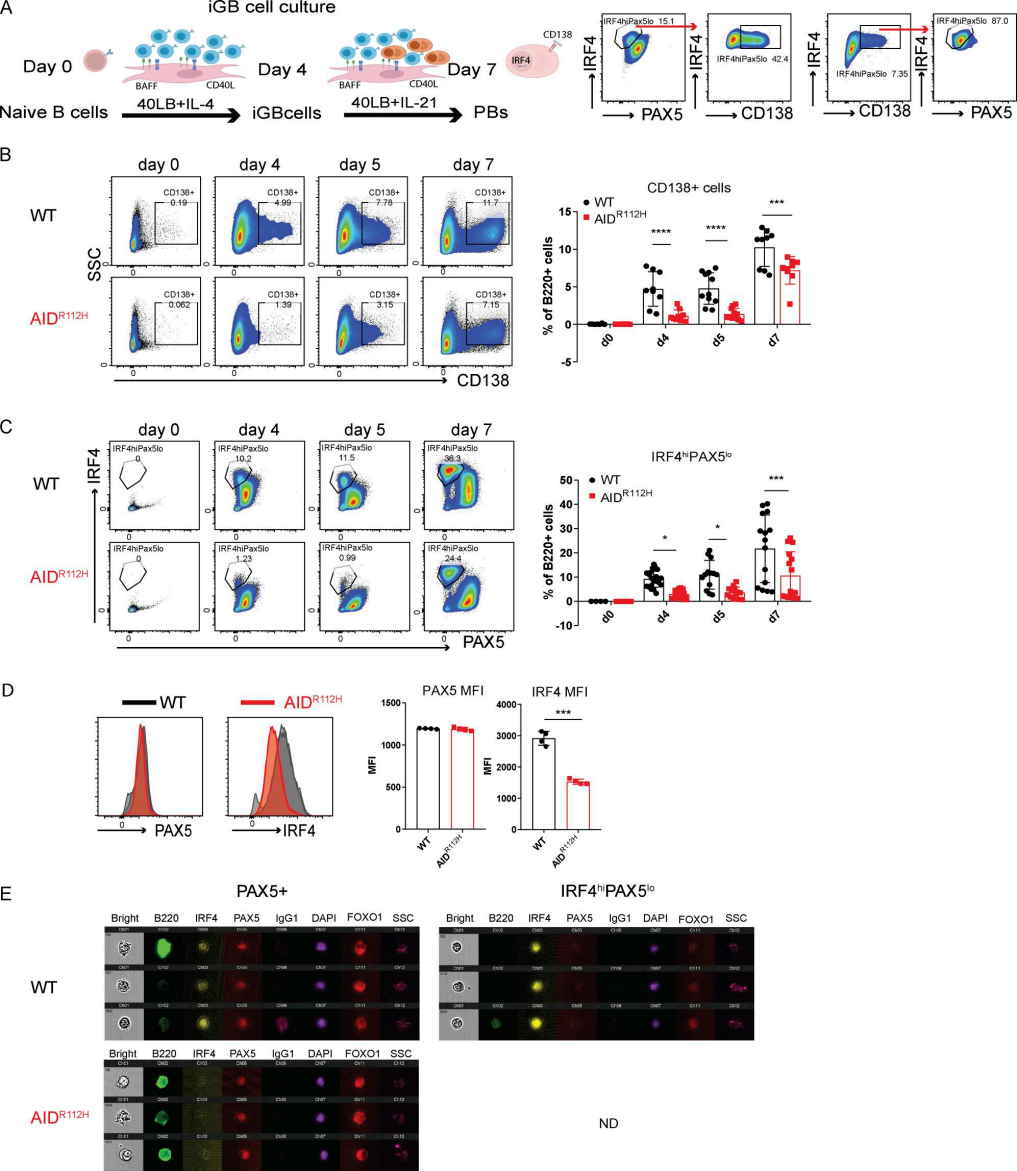
**Reduced AID** ^**R112H**^ **PC** **formation from in vitro iGB cells. (A)** PC differentiation in iGB cultures. Schematics of B cells cultured on 40LB cells and representative FACS plots for PB differentiation (CD138^+^ and IRF4^hi^PAX5^lo^ cell population). **(B)** CD138^+^ PB cells from day 0 to day 7. FACS plots and quantification, day 4 (P < 0.0001), day 5 (P < 0.0001), and day 7 (P = 0.0009) (*n* = 9–11, four experiments). **(C)** IRF4^hi^PAX5^lo^ cells from day 0 to day 7. FACS plots and quantification, day 4 (P = 0.016), day 5 (P = 0.033), and day 7 (P = 0.0001) (*n* = 13–22, five experiments). **(D)** Expression of IRF4 and PAX5 of iGB cells. Overlay of histograms of IRF4 and PAX5 acquired by flow cytometry. Quantification, IRF4 MFI: P = 0.00082, nonsignificant difference for PAX5 MFI, unpaired *t* test (*n* = 4, one representative experiment). **(E)** ImageStream analysis of IRF4, PAX5, and FOXO1 in iGB cells on day 7. Representative images of PAX5^+^ and IRF4^hi^PAX5^lo^ cells from the iGB culture. Images were acquired with ImageStream flow cytometry (10,000 cells acquired, two experiments). ND, nondetectable (no cells could be detected). Value and error bar: mean ± SD. *P < 0.05, ***P < 0.001, ****P < 0.0001.

To investigate whether the defective PC differentiation in AID^R112H^ mice is B cell–intrinsic, we examined PC differentiation using the iGB cell culture system (Fig. 5 A) (Nojima et al., 2011). Similar to PCs generated in vivo, B cells differentiating into PBs downregulated PAX5 and upregulated IRF4 and CD138 (Fig. 5 A). On day 7, a substantial proportion of cells had differentiated into PBs in the WT cell culture (Fig. 5, B and C). Consistent with our in vivo data, the differentiation of PBs from AID^R112H^ B cells was significantly compromised (Fig. 5, B and C). AID^R112H^ iGB cells showed reduced IRF4 protein when compared to WT iGB cells, while PAX5 protein was similar in WT and AID^R112H^ iGB cells (Fig. 5 D). The impaired upregulation of IRF4 in AID^R112H^ iGB cells was not due to compromised cell proliferation (Fig. S2 F). Analysis of iGB cells using imaging flow cytometry confirmed that AID^R112H^ B cells had greatly reduced nuclear IRF4 when compared to WT B cells (Fig. 5 E). At the same time, PAX5 and FOXO1, two transcription factors that are present in the GC B cells and downregulated in PCs (Nera et al., 2006; Sander et al., 2015; Dominguez-Sola et al., 2015; Omori et al., 2006), were similar in WT and AID^R112H^ iGB cells, and indeed were downregulated in the WT IRF4^hi^PAX5^lo^ population (Fig. 5 E).

We next examined whether ectopic expression of IRF4 could rescue defective PB generation in AID^R112H^ iGB cells. The overexpression of IRF4 in the AID^R112H^ iGB cells by retroviral transfection was able to fully restore the IRF4^hi^PAX5^lo^ cell population (Fig. S2 G), showing that upregulation of IRF4 plays a critical role in promoting PC differentiation. Moreover, upregulation of IRF4 may represent one of the earliest detectable events of PC fate–committed B cells. IRF4^hi^PAX5^lo^ cells had an increased intracellular Ig production (Fig. S2 H), but not all IRF4^hi^PAX5^lo^ cells expressed surface marker CD138 (Fig. 5 A and Fig. S2 H).

These results validated our in vivo findings and showed that reduced PB generation in AID^R112H^ mice was caused by a B cell–intrinsic defect to upregulate IRF4.

### High-affinity BCR fails to rescue PC differentiation of AID^R112H^ GC B cells

**Figure 6.**
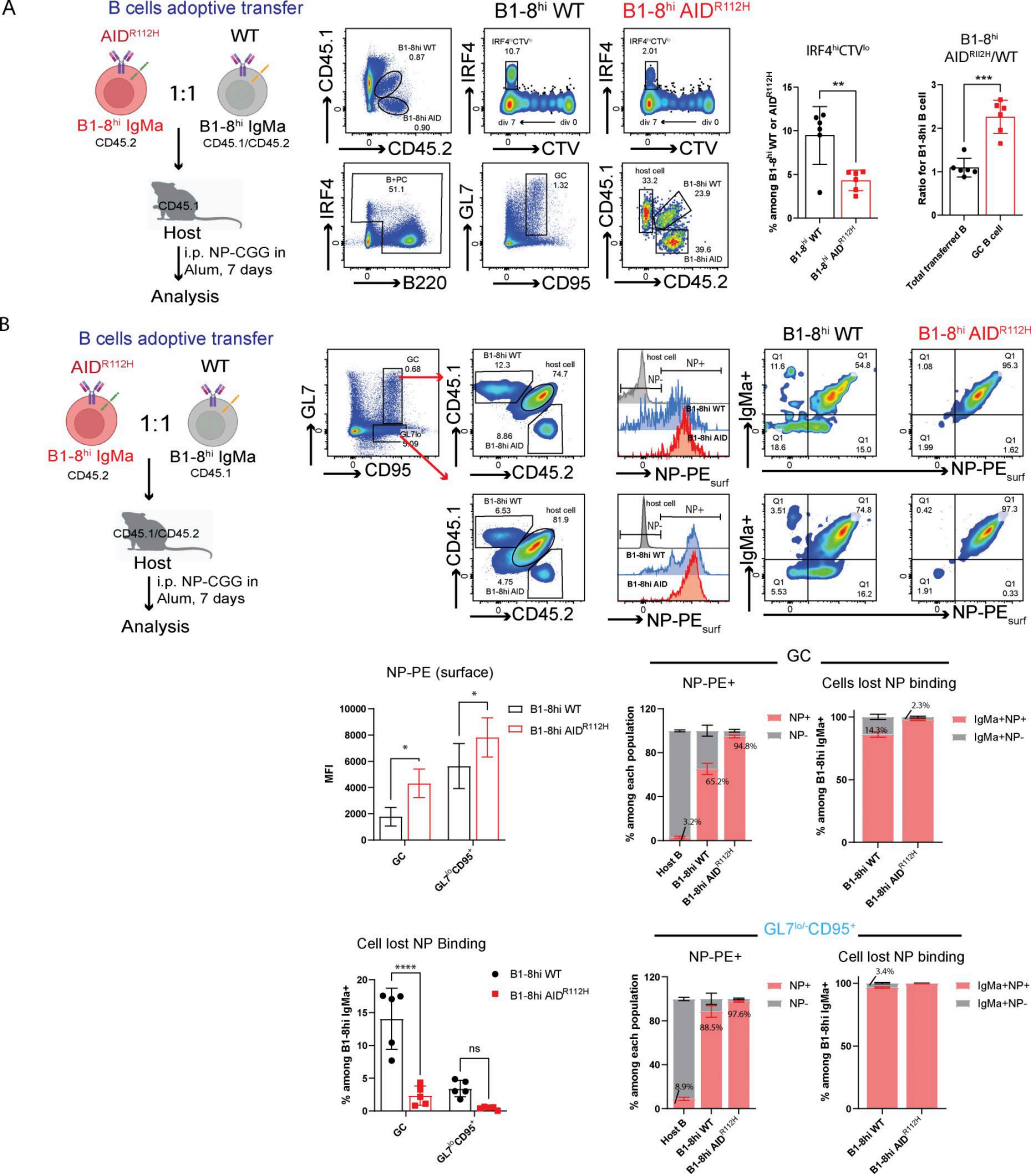
**High-affinity BCR is unable to rescue** **PC** **differentiation in AID** ^**R112H**^ **B cells. (A)** PC differentiation of B1-8^hi^WT and B1-8^hi^AID^R112H^ cells in vivo. Left: Experiment scheme. Middle: gating strategy to analyze GC response and PC differentiation from adoptively transferred B1-8^hi^WT and B1-8^hi^AID^R112H^ cells. Right: Quantification, IRF4^hi^CTV^lo^ in the adoptive transferred WT and AID^R112H^ cells: **P = 0.005; B1-8^hi^AID^R112H^/B1-8^hi^WT ratio in GC: ***P = 0.0009; paired *t* test (*n* = 6, two experiments). **(B)** NP binding capacity of B1-8^hi^ GC B cells. Upper: Gating strategy to detect the NP binding capacity of B1-8^hi^ B cells in GC by FACS. Middle: Left, surface NP-PE MFI in GC and GL7^lo^CD95^+^, B1-8^hi^WT versus B1-8^hi^AID cells: *P < 0.05, one-way ANOVA (*n* = 5, two experiments). Right: Fraction of GC B cells that were NP-PE^+^ and NP-PE^−^ among the host B, B1-8^hi^WT and B1-8^hi^AID B cells; fraction of BCR (IgMa)-expressing GC B cells that were NP-PE^+^ and NP-PE^−^ among B1-8^hi^WT and B1-8^hi^AID B cells. Lower: Left, quantification of IgM^+^ B1-8^hi^WT and B1-8^hi^AID cells that lost NP binding in GC and GL7^lo^CD95^+^ cells, ****P < 0.0001 for GC, nonsignificant for GL7^lo^CD95^+^, one-way ANOVA (*n* = 5, two experiments). Right: Fraction of GL7^lo^CD95^+^ B cells that were NP-PE^+^ and NP-PE^−^ among the host B, B1-8^hi^WT, and B1-8^hi^AID B cells; fraction of BCR (IgM)-expressing GL7^lo^CD95^+^ B cells that were NP-PE^+^ and NP-PE^−^ among B1-8^hi^WT and B1-8^hi^AID B cells.

**Figure S3.**
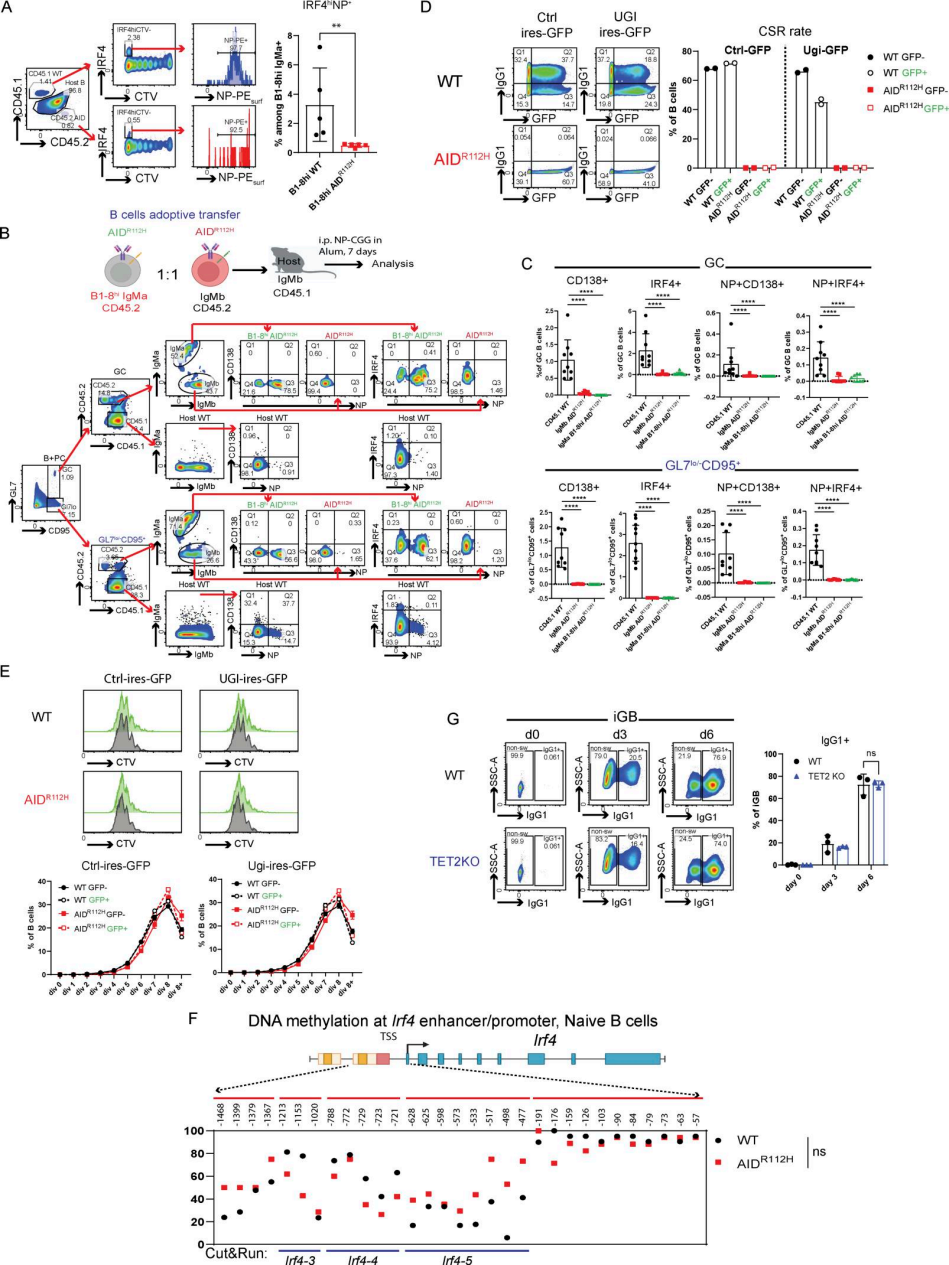
**High-affinity BCR failed to rescue** **PC** **differentiation and involvement of UNG and TET2 for IRF4 upregulation in GC B cells. (A)** In vivo generation of IRF4^hi^NP^+^ cells from B1-8^hi^WT and B1-8^hi^AID cells. Left: Gating strategy to detect NP-specific (surface staining) IRF4^hi^ cells by flow cytometry. Right: Quantification, P < 0.01, *t* test (*n* = 5, two experiments). **(B)** Schematics of in vivo experimental setup and gating strategy to analyze PC differentiation capacity of B1-8^hi^AID^R112H^ (green), AID^R112H^ (red), and CD45.1 WT (black) cells. Representative FACS plots for GC B cells and GL7^lo/−^CD95^+^ B cells. **(C)** Quantification of CD138^+^, IRF4^+^, NP^+^CD138^+^, and NP^+^IRF4^+^ cells in GC and GL7^lo/−^CD95^+^ cells. CD45.1 WT versus CD45.2 AID^R112H^: P < 0.0001 for all groups; CD45.1 WT versus CD45.2 B1-8^hi^AID^R112H^: P < 0.0001 for all groups, one-way ANOVA (*n* = 9, two experiments). Value and error bar: mean ± SD. **(D)** Reduced Ig class switching to IgG1 upon UGI-IRES-GFP retroviral transfection of iGB cells. Upper: Representative FACS plots for control plasmid and UGI-expressing plasmid transfection and Ig switching. Lower: Quantification of Ig switching to IgG1 among the control plasmid and UGI plasmid–transfected and nontransfected cells. **(E)** iGB cell proliferation rate upon UNG inhibition by UGI. Upper: Histogram overlay for nontransfected (gray) versus transfected (green) iGB cells (left: control plasmid; right: UGI plasmid). Lower: Quantification of the percentage of cells in each division in the transfected and nontransfected cells for both WT and AID^R112H^ iGB. Data are from one representative experiment with replicates. **(F)** CpG DNA methylation status at the *Irf4* locus in naive B cells from WT and AID^R112H^ mice. Top: Schematics for the *Irf4* gene locus and the region examined for DNA methylation using bisulfite sequencing. DNA methylation level at CpG sites, number above the graph shows the CpG position relative to first exon. For each CpG site, 15–30 clones were analyzed; data are from two independent experiments. **(G)** Ig class switching of WT and TET2 KO iGB cells on day 3 and day 6. Upper: representative FACS plots; lower: quantification, nonsignificant, ANOVA (*n* = 3, one experiment). **P < 0.01, ****P < 0.0001.

To examine whether the defective PC differentiation of AID^R112H^ GC B cells was caused by compromised affinity maturation, we took advantage of the B1-8^hi^ mice carrying a preassembled Ig heavy chain that when paired with an Ig lambda chain produce a BCR with high affinity for NP. To compare the NP high-affinity WT and AID^R112H^ B cells side by side, we adoptively transferred CTV-labeled naive B cells from B1-8^hi^AID^R112H^ and B1-8^hi^WT mice mixed at a 1:1 ratio and analyzed at day 7 upon immunization with NP-CGG (Fig. 6 A). B1-8^hi^AID^R112H^ and B1-8^hi^WT B cells maximally diluted CTV, suggesting an equivalent cell proliferation. IRF4^hi^ PBs were among the most divided cell population (≥7 divisions) from both B1-8^hi^AID^R112H^ and B1-8^hi^WT B cells in vivo (Fig. 6 A). There was a more than twofold reduction of IRF4^hi^ PBs from B1-8^hi^AID^R112H^ B cells as compared to B1-8^hi^WT B cells (Fig. 6 A). Moreover, there was more than threefold reduction of antigen-specific PCs (IRF4^hi^NP^+^) generated from B1-8^hi^AID^R112H^ cells compared with B1-8^hi^WT B cells (Fig. S3 A), suggesting an impaired high-affinity antibody response. Analysis of the GC response revealed that the GC B cell population was composed of both host B cells and adoptively transferred B1-8^hi^AID^R112H^ and B1-8^hi^WT B cells. Although the total transferred B1-8^hi^AID^R112H^ and B1-8^hi^WT B cells maintained a 1:1 ratio 7 days after immunization, there was a more than twofold accumulation of B1-8^hi^AID^R112H^ B cells over B1-8^hi^WT B cells in the GC (Fig. 6 A).

To further validate this, we adoptively transferred naive B cells from B1-8^hi^AID^R112H^ (IgMa, CD45.2) and AID^R112H^ (IgMb, CD45.2) mice, mixed at a 1:1 ratio and injected into WT recipient mice (IgMb, CD45.1, Fig. S3 B). At day 7 upon NP-CGG immunization, the PC differentiation from B1-8^hi^AID^R112H^ (IgMa, CD45.2) GC B cells was comparable to the differentiation from AID^R112H^ (IgMb, CD45.2) GC B cells, and both were significantly lower than that from WT (IgMb, CD45.1) GC B cells (Fig. S3, B and C).

In the adoptive transfer model, we observed a higher percentage of NP^+^ cells among the B1-8^hi^AID^R112H^ GC B population when compared to the NP^+^ cells among the B1-8^hi^WT GC B cells (94.8% versus 65.2%, Fig. 6 B). There was an increased MFI of surface NP-PE binding of B1-8^hi^AID^R112H^ GC B cells compared with the B1-8^hi^WT cells. This was likely due to both an increased fraction of NP^+^ B cells and a higher BCR level of AID^R112H^ GC B cells (Stewart et al., 2018). A fraction of IgM-expressing cells contained BCR that had lost NP binding in B1-8^hi^WT GC B cells, which was not observed in the B1-8^hi^AID^R112H^ GC B cells (14.2% versus 2.3%, Fig. 6 B). This can be attributed to SHM mediated by AID in the WT cells.

Together, these data suggest that the defective upregulation of IRF4 and PC differentiation of AID^R112H^ B cells did not result from impaired affinity maturation.

### Restored AID activity rescues PC differentiation

**Figure 7.**
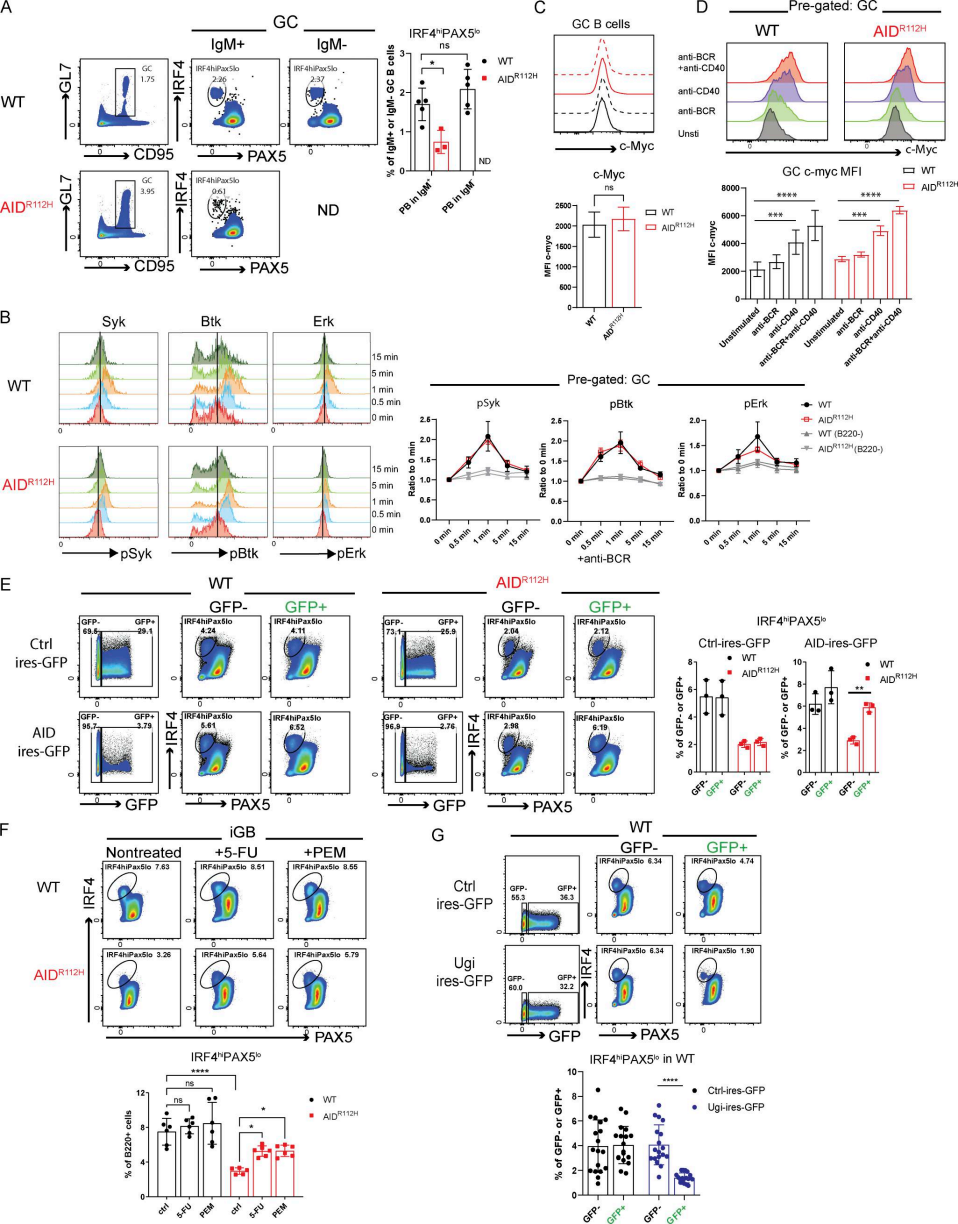
**PC** **differentiation requires** **WT** **AID protein. (A)** PC differentiation capacity of switched (IgM^−^) and nonswitched (IgM^+^) GC B cells. Left: Representative FACS plots. Right: Quantification, WT versus AID^R112H^, PB in IgM^+^, *P = 0.0102; IgM^+^ versus IgM^−^ PB in WT GC, nonsignificant, unpaired *t* test (*n* = 3–5, one experiment). **(B)** BCR signaling in WT and AID^R112H^ GC B cells. Left: FACS plot histogram overlay at different time points upon anti-BCR challenge. Right: Quantification, WT versus AID^R112H^, nonsignificant, unpaired *t* test (*n* = 4, two experiments). **(C)** c-MYC MFI in GC B cells. Right: Histogram overlays to show WT (black, solid and dotted line) and AID^R112H^ (red, solid and dotted line) cells, *t* test (*n* = 9, three experiments). **(D)** Upregulation of c-MYC upon stimulation for 2 h in GC B cells. Left: Histogram overlay of unstimulated and stimulated GC B cells. Right: Quantification of c-MYC MFI, anti-BCR versus unstimulated, nonsignificant; anti-CD40 versus unstimulated, ***P < 0.001 for both WT and AID^R112H^; anti-CD40+anti-BCR versus unstimulated, ****P < 0.0001 for both WT and AID^R112H^; nonsignificant between WT and AID^R112H^ for corresponding stimulation conditions. Two-way ANOVA (*n* = 4 mice per condition, two experiments). **(E)** Rescue of PB differentiation by expressing WT AID protein in AID^R112H^ iGB cells. FACS plots and quantification, Ctrl-IRES-GFP: no changes between GFP^+^ versus GFP^−^ in WT and AID^R112H^ iGB cells; AID-IRES-GFP: nonsignificant between WT GFP^−^ versus WT GFP^+^; **P = 0.0085 between AID^R112H^ GFP^−^ versus AID^R112H^ GFP^+^, two-way ANOVA (one representative experiment from three). **(F)** Rescue of PB differentiation upon 5-FU and PEM treatment in iGB culture. FACS plots and quantification, ****P < 0.0001, WT versus AID^R112H^; *P = 0.0133, AID^R112H^ versus AID^R112H^+5-FU; *P = 0.012, AID^R112H^ versus AID^R112H^ + PEM, one-way ANOVA (*n* = 6, three experiments). **(G)** Inhibition of PB differentiation by expressing Ugi peptide in WT iGB cells. FACS plots and quantification, Ctrl-IRES-GFP: no changes between WT GFP^−^ versus WT GFP^+^; Ugi-IRES-GFP: P < 0.0001 between WT GFP^−^ versus WT GFP^+^, unpaired *t* test (*n* = 18, two experiments).

To rule out that the defective PC differentiation is caused by compromised Ig class switching of AID^R112H^ GC B cells, we analyzed the PC output rate in nonswitched (IgM^+^) and switched (IgM^−^) GC B cells from SRBC-immunized mice. Indeed, there was a comparable level of PC differentiation between IgM^−^ and IgM^+^ GC B cells in WT mice, while the IgM^+^ GC B cells from AID^R112H^ mice had decreased capacity to form PCs as compared to IgM^+^ B cells from WT mice (Fig. 7 A). Additionally, the capacity of BCR signaling was unaltered in AID^R112H^ GC B cells when compared to WT GC B cells as assessed by p-Syk, p-Btk, and p-Erk (Fig. 7 B). Flow cytometry analysis showed a comparable level of c-MYC expression in AID^R112H^ GC B cells compared with WT cells (Fig. 7 C). Upon stimulation, AID^R112H^ GC B cells upregulated c-MYC to a comparable level of WT GC B cells (Fig. 7 D). These data demonstrate that the defective upregulation of IRF4 and PC differentiation of AID^R112H^ GC B cells did not result from impaired affinity maturation, Ig class switching, and BCR-induced upregulation of c-MYC, indicating that there is another layer of IRF4 regulation mediated by AID activity.

To investigate whether AID activity is directly involved in orchestrating PC differentiation besides its role in affinity maturation during the GC response, we took advantage of the iGB culture system supplemented with IL-21, which provides signals to support PC differentiation and bypasses the BCR affinity-mediated selection (Nojima et al., 2011). We expressed WT AID protein in the AID^R112H^ iGB cells by retroviral transduction. AID^R112H^ cells that received the WT AID protein completely restored the IRF4^hi^PAX5^lo^ cell population when compared to AID^R112H^ cells that received a control plasmid (Fig. 7 E). This establishes that WT AID activity plays a critical role in the PC fate decision in addition to its role in affinity maturation–driven selection.

To mimic the molecular consequence of AID deamination activity, we used 5-fluorouracil (5-FU) and pemetrexed (PEM) that elevate the level of deoxyuridine triphosphate and lead to incorporation of deoxyuridine into DNA (Longley et al., 2003). 5-FU– and PEM–treated WT iGB cells had no change in generation of the IRF4^hi^PAX5^lo^ cell population when compared to the nontreated WT cells. In contrast, the AID^R112H^ iGB cells treated with 5-FU and PEM had increased IRF4^hi^PAX5^lo^ populations when compared to nontreated AID^R112H^ cells (Fig. 7 F). This prompted us to investigate whether UNG that senses and removes uracil from DNA is involved in regulation of PC differentiation. We ectopically expressed Ugi, a small peptide inhibitor, in WT iGB cells to inhibit UNG activity (Di Noia and Neuberger, 2002). Reduced Ig CSR in Ugi-transduced cells suggested efficient inhibition of UNG activity, while the cell proliferation rate was unaffected in the Ugi-transduced cells compared with the control plasmid–transduced iGB cells (Fig. S3, D and E). WT cells that received the Ugi reduced the IRF4^hi^PAX5^lo^ cell population by ≥50% when compared to cells that received a control plasmid (Fig. 7 G).

These data suggest that the deamination activity of AID and the signaling cascade elicited by DNA U:G mismatch were involved in upregulation of IRF4 for PC fate decision in the GC B cells.

### AID accelerates demethylation of the *Irf4* enhancer/promoter via cooperation with TET2 in GC B cells

**Figure 8.**
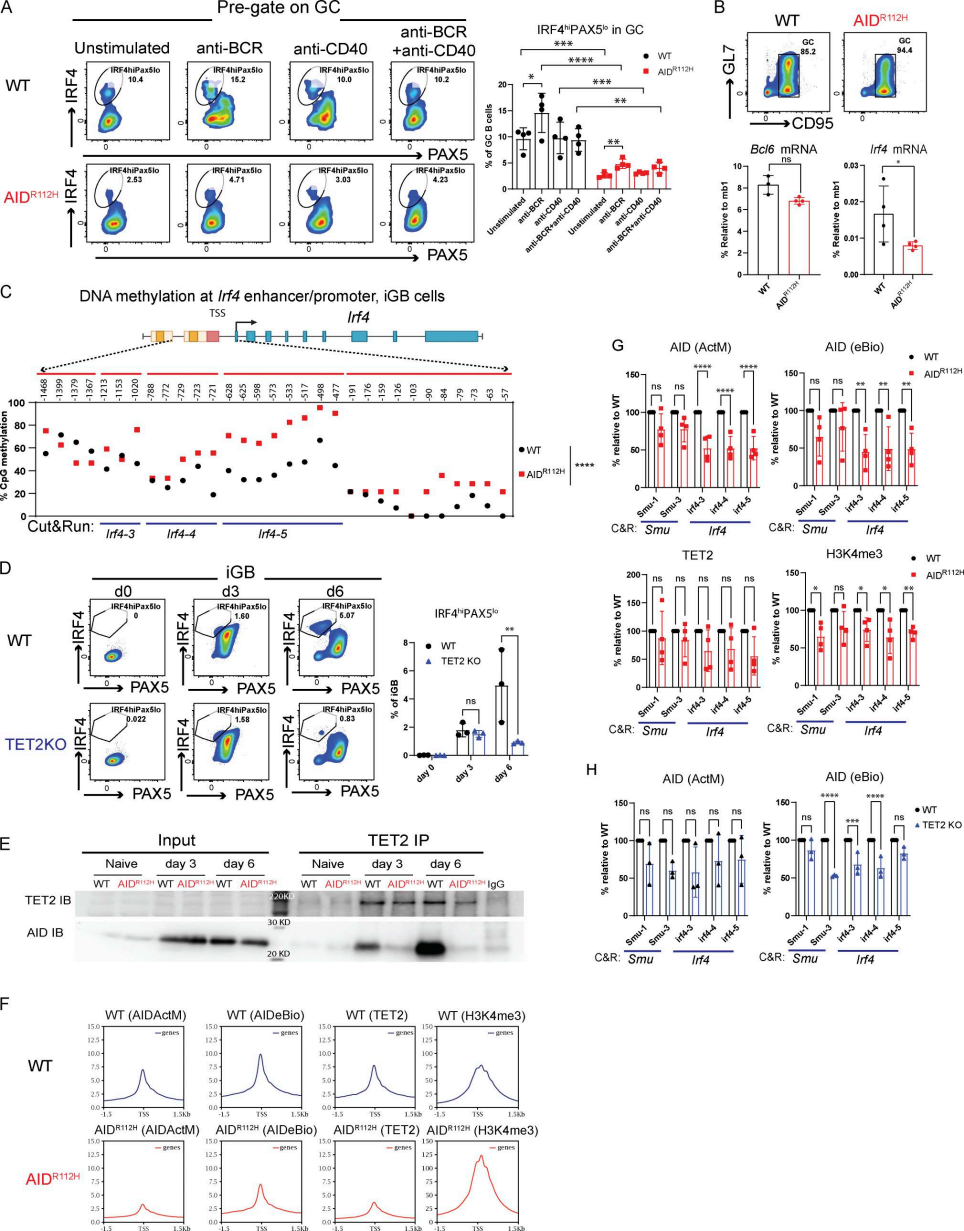
**AID cooperates with TET2 for efficient *Irf4* enhancer/promoter demethylation in GC B cells. (A)** Percentage of IRF4^hi^PAX5^lo^ cells in GC at day 7 and upon stimulation for 2 h. Left: Representative FACS plots (pregated on GC B cells). Right: Quantification, WT, anti-BCR versus unstimulated, P < 0.05 (*t* test), nonsignificant for other stimulations; AID^R112H^, anti-BCR versus unstimulated, P < 0.01 (*t* test), nonsignificant for other stimulations. Two-way ANOVA between genotypes (*n* = 4 per condition, two experiments). **(B)***Bcl6* and *Irf4* mRNA level in purified GC B cells. Upper panels: GC purity by flow cytometry. Lower panels: Quantification of *Bcl6* and *Irf4* mRNA level by RT-PCR; each dot represents an average value from an individual animal (technical triplicates for each animal), *Bcl6* mRNA, WT versus AID^R112H^, nonsignificant; *Irf4* mRNA, WT versus AID^R112H^, P < 0.05, ratio-paired *t* test (*n* = 3–4, two experiments). Value and error bar: mean ± SD. **(C)** CpG DNA methylation status at the *Irf4* locus in iGB cells derived from WT and AID^R112H^ cells on day 7. Top: Schematics for *Irf4* gene locus and the region examined for DNA methylation using bisulfite sequencing. DNA methylation level at CpG sites; number above the graph shows the CpG position relative to the first exon. For each CpG site, 15–30 clones were analyzed, WT versus AID^R112H^, P < 0.0001, paired *t* test, data from two independent experiments. **(D)** IRF4^hi^PAX5^lo^ cell differentiation in TET2 KO iGB cells. Representative FACS for day 0, day 3, and day 6. Quantification, day 6, WT versus TET2 KO, P < 0.01, two-way ANOVA (*n* = 3, one experiment). **(E)** Co-immunoprecipitation of endogenous AID and TET2 protein from iGB cells. Anti-TET2 pulldown and immunoblot to detect TET2 (upper panel) and AID (lower panel, uncropped blot image in Fig. S4 B in the same order). One representative blot from four independent experiments. **(F)** CUT&RUN sequencing to detect global chromatin association of AID, TET2, and H3K4me3 in iGB cells on day 3. Upper: Average enrichment profiles near TSS (−1.5 to +1.5 kb). **(G)** CUT&RUN and qPCR to detect association of AID, TET2, and H3K4me3 with the *Irf4* locus in iGB cells on day 3. CUT&RUN detection region *Irf4-3*, *Irf4-4*, and *Irf4-5* as indicated in the bottom of C. Black column: WT; red column: AID^R112H^; y axis: % relative to WT, *P < 0.05, **P < 0.01, anti-AID (Active Motif and eBioscience), anti-TET2, anti-DNMT1 and anti-H3K4me3, two-way ANOVA (*n* = 4, four experiments). **(H)** CUT&RUN and qPCR to detect association of AID with the Switch mu region and the *Irf4* enhancer/promoter in TET2 KO iGB cells on day 3. Black column: WT; blue column: TET2 KO; y axis: % relative to WT, ***P < 0.001, ****P < 0.0001, two-way ANOVA (*n* = 3, three experiments). Source data are available for this figure: SourceData F8.

Recent work has demonstrated that the level of the IRF4 protein was restricted posttranscriptionally by ubiquitin ligases Cbl and Cbl-b in GC B cells (Li et al., 2018). To understand whether the failure to upregulate IRF4 in AID^R112H^ GC B cells occurred at the transcriptional level or at the posttranscriptional level, we stimulated WT and AID^R112H^ GC B cells in vitro and examined the IRF4^hi^Pax5^lo^ population. WT GC B cells upregulated IRF4^hi^PAX5^lo^ cells upon BCR activation, whereas AID^R112H^ GC B cells failed to upregulate the IRF4^hi^PAX5^lo^ population (Fig. 8 A). The overall level of the IRF4^hi^PAX5^lo^ population among AID^R112H^ GC B cells was reduced compared with WT GC B cells in both unstimulated and stimulated groups, suggesting a lower mRNA level of *Irf4* in AID^R112H^ GC B cells. We next examined the *Irf4* mRNA level from enriched GC B cells by quantitative (q)PCR. The mRNA level of *Irf4* from AID^R112H^ GC B cells showed a twofold reduction, in contrast to the *Bcl6* mRNA that remained comparable between AID^R112H^ and WT GC B cells (Fig. 8 B).

**Figure S4.**
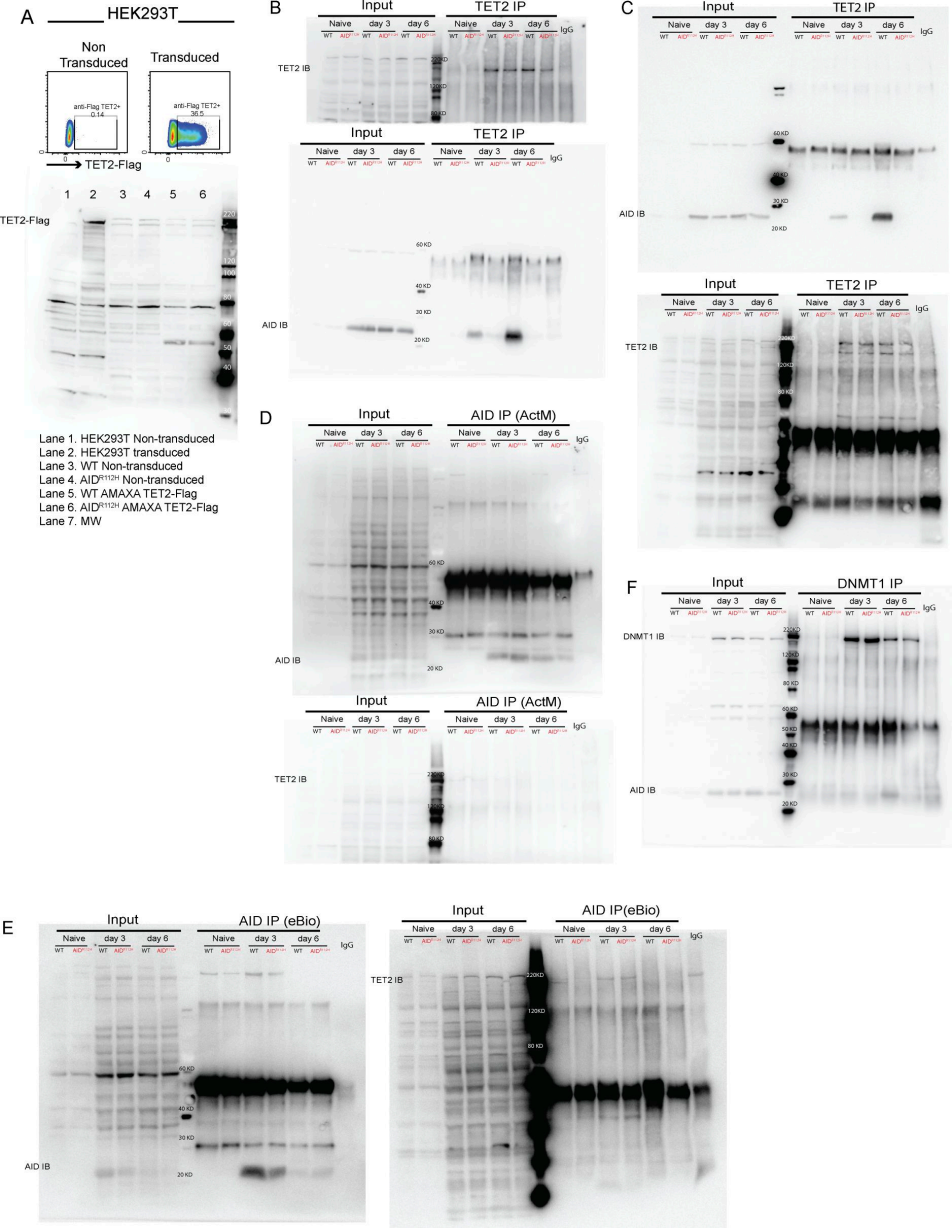
**Co-immunoprecipitation of AID, TET2, and DNMT1 in iGB cells. (A)** Validation of TET2 antibody by western blot in TET2-transduced and nontransduced cells. Upper: FACS plot for the control and TET2-Flag–transduced HEK293T cells. Lower: Western blot to detect TET2. Lane 1: Nontransduced HEK293T cells; lanes 3–6: WT and AID^R112H^ iGB cells underwent TET2 transfection by AMAXA electroporation; Lane 7: protein ladder. **(B)** TET2 immunoprecipitation on day 3 and day 6. Immunoblot for TET2 (Abcam, upper panel) and AID (Active Motif, lower panel), cropped blot images shown in Fig. 8 E. **(C)** Repeat of TET2 immunoprecipitation on day 3 and day 6. Immunoblot for AID. **(D)** AID immunoprecipitation on day 3 and day 6 using anti-AID antibody (Active Motif). Immunoblot for AID (Active Motif, upper panel) and TET2 (Abcam, lower panel). **(E)** AID immunoprecipitation on day 3 and day 6 using anti-AID antibody (eBioscience). Immunoblot for AID (Active Motif, left panel) and TET2 (Abcam, right panel). **(F)** DNMT1 immunoprecipitation on day 3 and day 6. Immunoblot for AID (Active Motif) and DNMT1 (Merck). Source data are available for this figure: SourceData FS4.

**Figure S5.**
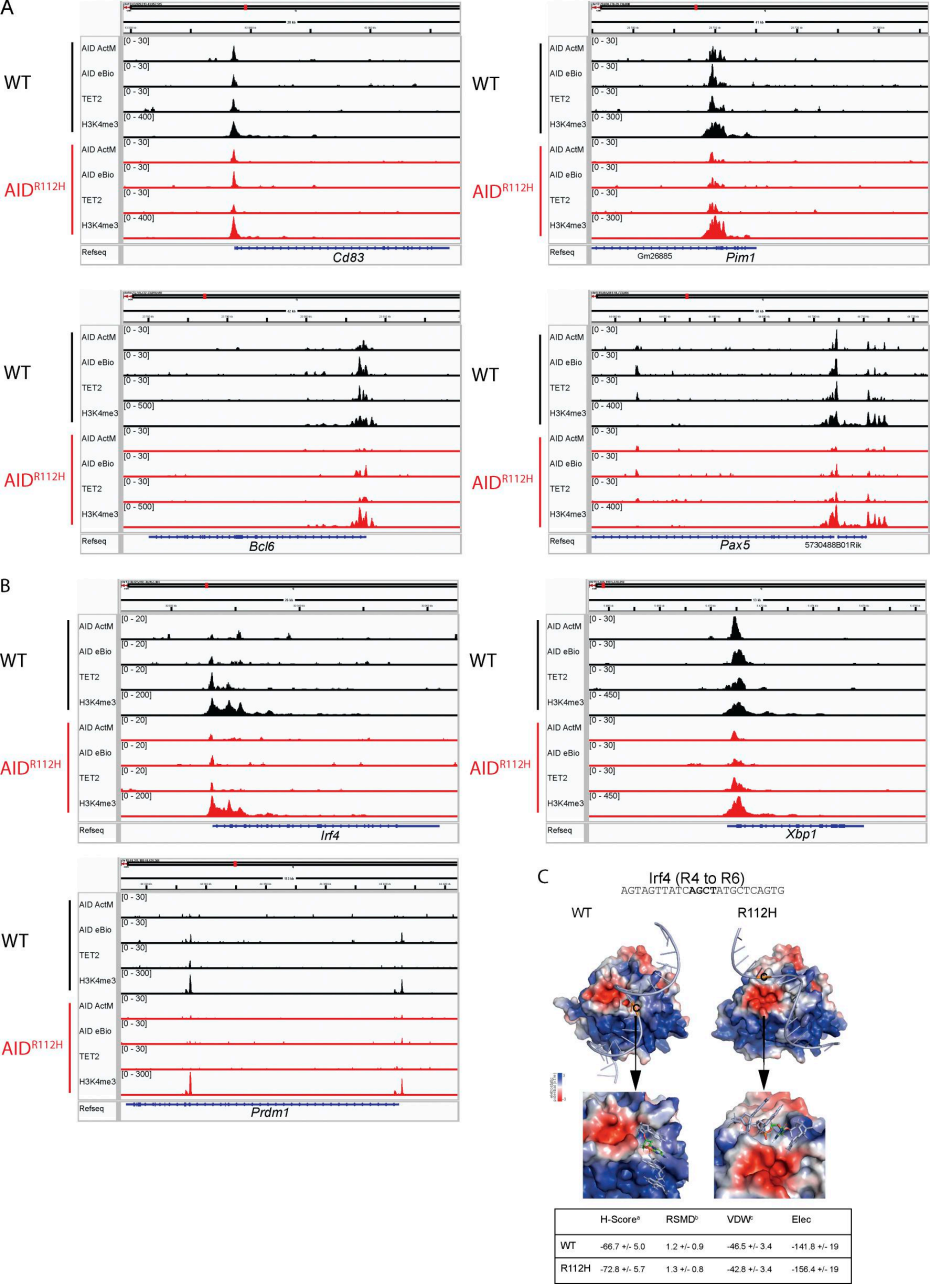
**Association of AID, TET2, and histone mark H3K4me3 with AID off-target genes. (A)** CUT&RUN-sequencing peak profiles of genes involved in the GC response including *Cd83*, *Pim1*, *Bcl6*, and *Pax5.***(B)** CUT&RUN-sequencing peak profiles for genes involved in PC differentiation including *Irf4*, *Xbp1*, and *Prdm1*. The enrichment profiles represent the mean of two separate experiments sequenced at the same time. **(C)***Irf4* probe binding to AID active-site cleft. Left upper: *Irf4* probe binding to WT and R112H AID protein. Left lower: Zoomed views of the simulated docked complex. The AGCT motif is displayed as licorice representation, and cytosine is colored in green (carbon atoms). Right: The values for the top-ranked structures. The values were obtained from HADDOCK software. a, HADDOCK score. b, RMSD (Å) value from the overall lowest-energy structure. c, van der Waals energy (kcal/mol). d, electrostatic energy (kcal/mol).

Upregulation of IRF4 is associated with a progressive DNA demethylation at the *Irf4* enhancer/promoter region as B cells transit through the GC to form PCs (Fujii et al., 2020). Recent studies have demonstrated a role of AID in regulating the DNA methylome in GC B cells (Dominguez et al., 2015; Català-Moll et al., 2021). To test whether the impaired *Irf4* transcription was due to compromised DNA demethylation in AID^R112H^ GC B cells, we analyzed the DNA methylation pattern at the *Irf4* enhancer/promoter region (−1,471 to −14 bp relative to the first exon) using bisulfite sequencing. This indeed revealed an impaired DNA demethylation at the *Irf4* enhancer/promoter in AID^R112H^ iGB cells when compared to WT iGB cells on day 7 (Fig. 8 C and Fig. S3 F). TET proteins play a critical role in mediating demethylation of the *Irf4* enhancer/promoter during B cell differentiation into PCs (Fujii et al., 2020). Intriguingly, overall transcriptome and methylome alterations occurring in TET2 knockout (TET2 KO) GC B cells are largely shared by AID knockout GC B cells (Rosikiewicz et al., 2020). Therefore, we reasoned that AID and TET2 may cooperate to mediate *Irf4* demethylation. Indeed, TET2 KO iGB cells showed a reduction of IRF4^hi^PAX5^lo^ cells compared with WT iGB cells on day 6 (Fig. 8 D). The reduced IRF4^hi^Pax5^lo^ population did not result from the lower level of the AID protein in the TET2 KO iGB cells, because these cells underwent a comparable level of Ig class switching as WT iGB cells on both day 3 and day 6 (Fig. S3 G) (Lio et al., 2019). To examine a possible interaction between AID and TET2 proteins in B cells, we performed co-immunoprecipitation using iGB cells from day 3 and day 6 cultures. We first confirmed the specificity of the TET2 detection by expressing Flag-tagged TET2 in HEK293 cells (Fig. S4 A). We detected AID from TET2 immunoprecipitations in WT iGB cells but not in the AID^R112H^ iGB cells (Fig. 8 E; and Fig. S4, B and E). AID was undetectable from the DNMT1 immunoprecipitation from either WT or AID^R112H^ iGB cells (Fig. S4 F). This suggested a proximity of AID and TET2 proteins at the chromatin level in WT iGB cells, which was compromised in AID^R112H^ iGB cells. To examine the AID- and TET2-associated genomic loci in iGB cells, we performed Cleavage Under Targets and Release Using Nuclease (CUT&RUN) sequencing and qPCR. Results from the CUT&RUN sequencing revealed a preferential association of AID with the promoter region of genes (Fig. 8 F). Association of AID and TET2 to chromatin at the global level was reduced in AID^R112H^ iGB cells compared with WT cells as confirmed using two different AID antibodies (Fig. 8 F). A closer examination at several selected gene loci revealed genes with combined reduction of both AID and TET2 chromatin association (*Bcl6*, *Pax5*, and *Pim1*), genes with reduction of either AID or TET2 chromatin association (*Cd83*, *Irf4*, and *Xbp1*)*,* and genes with minimum chromatin association of AID or TET2 (*Prdm1*, Fig. S5, A and B). In contrast, the global level of H3K4me3 spanning the transcription start site (TSS) had a moderate increase in the AID^R112H^ iGB cells compared with WT cells (Fig. 8 F). To further investigate this in detail, we performed qPCR using CUT&RUN samples at the selected regions spanning the *Irf4* enhancer/promoter. The results showed a reduced association of AID with the *Irf4* enhancer/promoter in AID^R112H^ cells when compared to WT iGB cells on day 3 (Fig. 8 G). TET2 association was comparable in WT and AID^R112H^ iGB cells (Fig. 8 G). On the other hand, WT AID was associated with the *Irf4* enhancer/promoter in TET2 KO iGB cells (Fig. 8 H). Association of AID with Switch mu regions was comparable between AID^R112H^ and WT iGB cells, suggesting differential recruitment mechanisms of AID to non-Ig loci versus Ig loci. Interestingly, although there was a moderate increase of active histone mark H3K4me3 at the global level in AID^R112H^ iGB cells when compared to WT iGB cells, the H3K4me3 at the *Irf4* enhancer/promoter region showed a reduction in AID^R112H^ cells, indicating a reduced *Irf4* transcription initiation/elongation (Fig. 8, F and G).

Together, these data suggest that chromatin association of AID and TET2 was likely independent of each other. However, a synergistic effect from TET2 and AID led to efficient demethylation of the *Irf4* promoter/enhancer and that the absence of either TET2 or AID activity interferes with demethylation of the *Irf4* gene to induce transcription.

### In silico structure modeling of AID reveals how R112H abolishes the deaminase activity

**Figure 9.**
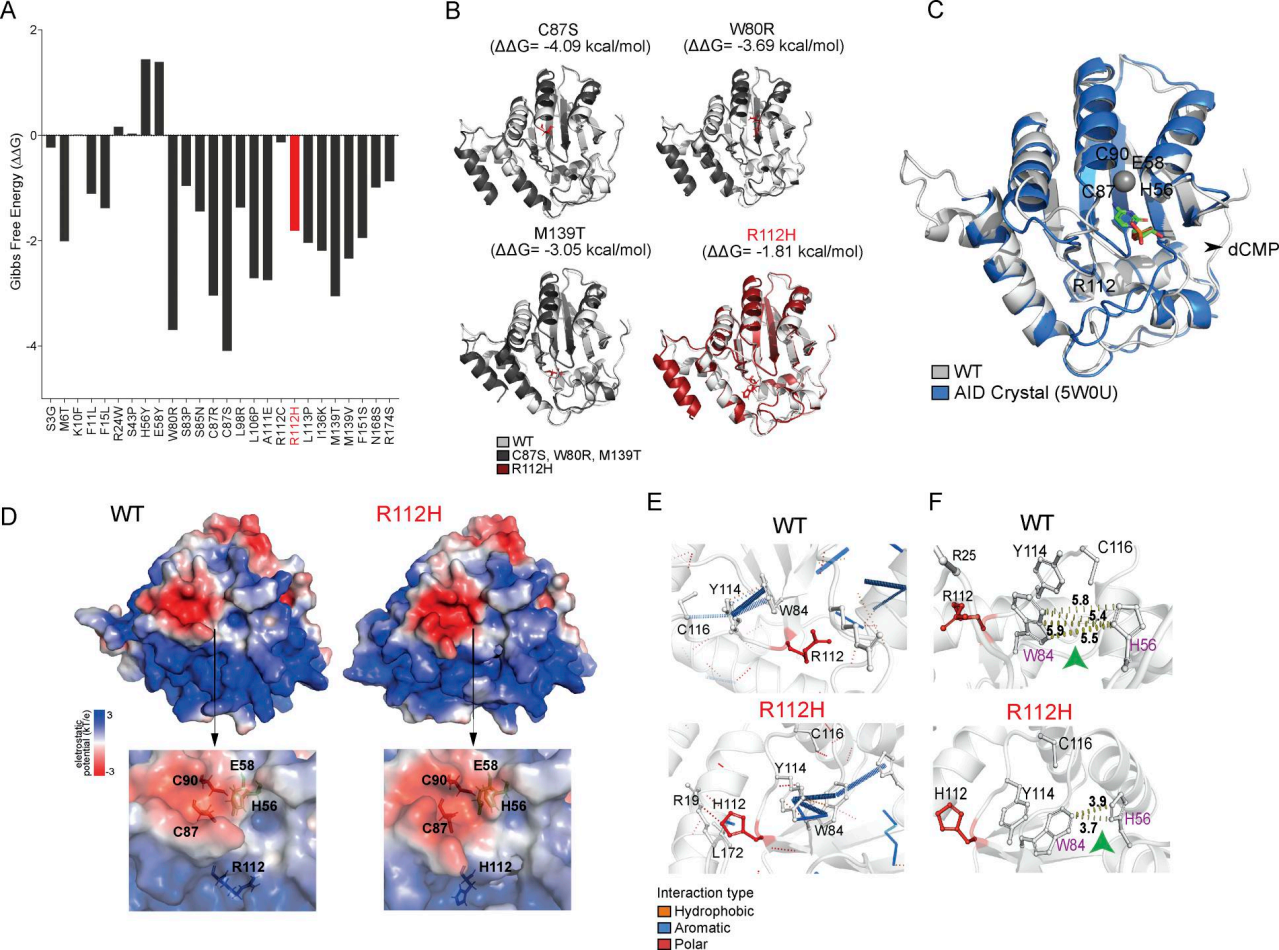
**Structure analysis of AID mutations reveals altered substrate binding pocket of AID** ^**R112H**^ **. (A)** DDMut scores of selected mutations in AID. Red marks R112H mutation; black marks other AID mutations. **(B)** 3D models of WT and mutated AID (predicted) aligned together. **(C)** Structure of AID from RoseTTAFold aligned to AID crystal bound to dCMP (PDB: 5W0U). Catalytic residues H56, E58, C87, and C90, and the residue R112 highlighted. **(D)** Electrostatic surface potentials of WT AID and AID^R112H^. Upper: whole protein; lower: catalytic pocket is zoomed in. Blue: positively charged residues; white: neutral residues; red: negatively charged residues. Position of primary catalytic residues H56, E58, C87, C90 together with R112 are highlighted. **(E)** Interatomic interactions of affected residues in WT AID and AID^R112H^. **(F)** Interaction distance between W84 and H56 in WT AID and AID^R112H^. Amino acids W84 and H56 are highlighted in purple. Green arrowheads highlight the interaction distance between the two highlighted amino acids. WT: 5.4–5.9 Å; AID^R112H^: 3.7–3.9 Å.

To examine the impact of the R112H mutation on protein structure and deamination activity of AID, we performed in silico modeling analysis. The AID RoseTTAFold structures were used to predict ΔΔG of selected mutations (Fig. 9, A–C). Among 26 patient mutations in AID, the R112H mutation showed an intermediate score (−1.81 kcal/mol) in contrast to the strongly destabilizing mutations, C87S (−4.09 kcal/mol), W80R (−3.69 kcal/mol), and M139T (−3.05 kcal/mol) (Fig. 9, B and C). The low root-mean-square deviation (RMSD) values after the alignment of the structures of C87S (0.425 Å), W80R (0.370 Å), M139T (0.372 Å), and R112H (0.441 Å) indicated minimal structural difference from the WT AID structure (Fig. 9 B). The H112 residue in AID^R112H^ indirectly altered the positive charge near the H56 and the negative charged surface around the catalytic pocket cytosines (Fig. 9 D). The R112H mutation induced a polar interaction to R19 and an aromatic interaction to L172, leading to reduced interaction distance between W84 and H56 (Fig. 9, E and F). This resulted in less accessibility to the catalytic pocket of mutant AID^R112H^ (King et al., 2015). Y114 had very high contact frequency with the phosphodiester backbone during ssDNA-AID binding (King et al., 2015), and Y114 interacted with an oxygen of dCMP (Qiao et al., 2017). The perturbed interaction between W84 with Y114 could indicate a change in the interaction between AID^R112H^ and ssDNA/dCMP. Indeed, molecular docking of AID^R112H^ with the *Irf4*-AGCT motif had a reduced electrostatic interaction energy compared with the WT-*Irf4*-AGCT complex (Fig. S5 C). Together, these data explain how the R112H mutation in the APOBEC-like domain of AID leads to a catalytically dead AID with a direct impact on the demethylation status of the *Irf4* gene.

## Discussion

Generation of high-affinity and long-lived PCs from GCs is essential for humoral immunity (Shlomchik and Weisel, 2012; Shapiro-Shelef and Calame, 2005). Using the AID^R112H^ mouse model that spontaneously develops GC hyperplasia (Dahlberg et al., 2014)*,* we here demonstrate that lack of AID activity halted GC B cell differentiation at a tGC and pre-PB stage, which resulted in the accumulation of GL7^lo/−^CD95^+^ B cells. The GL7^lo/−^ tGC B cells in AID^R112H^ mice failed to upregulate IRF4 irrespective of BCL6 downregulation and therefore were unable to initiate the PC differentiation program. The expression of a high-affinity BCR was unable to restore the halted PC generation from AID^R112H^ GC B cells in mice. Our data show that the synergistic effects of AID and TET2 accelerated the DNA demethylation of the *Irf4* enhancer/promoter and promoted *Irf4* transcription. This study identifies an important example where AID “off-target” activity has direct consequence for cell fate decision during B cell differentiation in GCs.

The cell fate decisions of B cells in GCs critically depend on the interplay between two groups of transcription factors (Shapiro-Shelef and Calame, 2005; Nutt et al., 2015). Downregulation of BCL6 precedes PC differentiation of GC B cells (Klein and Dalla-Favera, 2008; Shapiro-Shelef and Calame, 2005). Our study showed that the IRF4^hi^ population was nearly completely abolished among the BCL6^lo/−^ cells in AID^R112H^ mice. This suggests that downregulation of BCL6 alone was not sufficient for IRF4 upregulation and PC differentiation. These data strongly suggest an additional layer of regulation of IRF4 mediated by AID, independent of downregulation of BCL6 (Phan et al., 2007). Moreover, the GL7^lo/−^CD95^+^ cells in AID^R112H^ mice mainly consisted of halted tGC B cells as evident by the BCL6 expression. In WT mice, the GL7^lo/−^CD95^+^ cells were a heterogeneous cell population consisting of cells from both the GC pathway and the extrafollicular pathway. Therefore, the reduced IRF4^hi^PAX5^lo^ population in GL7^lo/−^CD95^+^ cells likely resulted from compromised generation of PCs from both the GC response and extrafollicular pathway. Importantly, the expression of the WT AID protein in the AID^R112H^ iGB cells fully restored generation of IRF4^hi^PAX5^lo^ cells. Treatment with 5-FU or PEM, which could increase the incorporation of deoxyuridine into DNA, partly restored the IRF4^hi^PAX5^lo^ cell population in AID^R112H^ iGB cells. Moreover, inhibition of UNG that removes uracil generated by AID using the small peptide inhibitor Ugi significantly decreased the IRF4^hi^PAX5^lo^ cells in WT iGB cultures, suggesting the involvement of UNG in PC fate decision downstream of AID-mediated deamination.

B cell deficiency for TET2 and TET3 leads to an increased GC response, aberrant accumulation of GC LZ B cells, and impaired PC generation, which resembles the GC response from the AID^R112H^ mice (Dominguez et al., 2018; Schoeler et al., 2019; this study). Our data demonstrate a collaboration between AID and TET2 in GC B cells, which provides an explanation for the phenotypic resemblance and the shared transcriptome and methylome changes observed in AID knockout and TET2 KO GC B cells (Rosikiewicz et al., 2020). Although there is still a controversy regarding a direct role of AID in active demethylation, altered DNA demethylation in GC B cells deficient for AID has been demonstrated by several studies in both mouse models and a hyper-IgM syndrome patient (Dominguez et al., 2015; Català-Moll et al., 2021). AID-associated loci are overrepresented in the hypomethylated loci of GC B cells (Shaknovich et al., 2011). Independent studies have shown that the purified AID protein can deaminate 5mCs in vitro although with less activity as compared to unmethylated cytosine deamination. It is unclear whether AID-dependent deamination on cytosines or 5mCs is directly involved in DNA demethylation. Moreover, to process TET2-mediated 5mC deoxygenation, cells employ a similar spectrum of repair proteins from the BER pathway as used for processing of AID-mediated cytosine deamination, e.g., TDG and apurinic/apyrimidinic endonuclease (Wu and Zhang, 2017). The synergistic effect of AID and TET2 may greatly augment the DNA damage response and facilitate the recruitment of the shared components from the BER pathway, which in turn accelerates the active demethylation of the *Irf4* enhancer/promoter. Similarly, incorporation of dUs in iGB cells by 5-FU/PEM treatment may trigger excessive amount of DNA damage that augments the recruitment of BER proteins and/or impaired maintenance of DNA methylation by DNMT1. This would lead to global DNA demethylation and increased IRF4^hi^Pax5^lo^ cells. Our data link the deamination activity of AID to epigenetic regulation during B cell differentiation.

The *Irf4* gene has been repeatedly identified as one of the AID off-target genes (Álvarez-Prado et al., 2018; Meng et al., 2014; Chiarle et al., 2011; Yamane et al., 2011). Our AID CUT&RUN data using two different AID antibodies suggested that AID preferentially associated with TSSs at its off-target genes, where TET2 showed shared occupancy. Although global chromatin association of AID and TET2 was reduced in AID^R112H^ B cells, the reduced association of both proteins was only observed at some off-target genes (*Bcl6*, *Pax5*, and *Pim1*). It is possible that chromatin association of AID and TET2 proteins is independent of each other. However, their synergistic effect may be important for efficient global methylome alteration during the differentiation from GC B cells to PCs. At the *Irf4* locus, our data show that this effect is translated into efficient DNA demethylation at the promoter/enhancer region. Our in silico modeling provided evidence that the R112H mutation affected the catalytic pocket of AID by indirectly altering the positive charge around H56 and the negative surface around the catalytic pocket. This would impact on the catalytic pocket accessibility, ssDNA binding, interaction with dCMP, and possibly the AID^R112H^ occupancy at on- and off-target sites.

AID off-targets at non-Ig loci are preferentially associated with B cell superenhancers undergoing convergent/divergent transcription (Meng et al., 2014; Qian et al., 2014; Pefanis et al., 2014). This predisposes the GC B cells into increased risk of malignant transformation, as chromosome translocation of IgH to proto-oncogenes is a hallmark of GC-derived B cell lymphomas (Casellas et al., 2016). Besides a few highly mutated off-target genes (e.g., *Bcl6*, *Pim1*), AID-dependent mutations at the majority of the off-target loci are usually neglectable due to the robust DNA repair in B cells (Liu et al., 2008; Yamane et al., 2011; Álvarez-Prado et al., 2018; Qian et al., 2014). It is possible that the AID-dependent deamination at some of these loci has been translated into epigenetic modification instead of mutations due to the availability of DNA repair factors and the epigenetic machinery at the loci. A central question concerns the mechanism that ensures robust SHM in GC B cells to generate high-affinity PCs, but that also prevents the heavily mutated GC B cells from undergoing tumor transformation. Here, we established that in addition to SHM and CSR, AID activity is critical to initiate the PC program in GCs by promoting IRF4 expression through locus-specific DNA demethylation. A mechanistic linkage of AID activity and downstream PC lineage commitment probably acts as an additional safeguard against adverse mutational effects of high-level SHM, as it would ensure their sequestration into a terminally differentiated and quiescent cell subset.

## Materials and methods

### Mice and cell transfers

Mice were bred in the animal facility of KM Wallenberg and Department of Microbiology, Tumor and Cell Biology, Stockholm, Sweden. All animal experiments performed are approved by the Stockholm North Animal Ethics Committee permits N272/14, 11159-18, and 05294-2023. AID^R112H^ C57BL/6 (Dahlberg et al., 2014) and WT C57BL/6 mice were littermates. To generate mixed bone marrow chimeric mice, 1 × 10^7^ mixed bone marrow cells in 1:1 ratio of CD45.1 WT cells and CD45.2 AID^R112H^ cells were injected intravenously (i.v.) to the lethally irradiated WT congenic recipient mice. B1-8^hi^ IgH knock-in mice were purchased from The Jackson Laboratory and crossed with AID^R112H^ mice to generate B1-8^hi^AID^R112H^ mice (IgMa, CD45.2) (Shih et al., 2002). 5 × 10^6^ naive spleen B cells mixed in 1:1 ratio from B1-8^hi^AID^R112H^ mice (IgMa, CD45.2) and AID^R112H^ mice (IgMb, CD45.2) were injected i.v. into WT recipient mice (IgMb, CD45.1). This was followed by i.p. immunization with 50 μg NP-CGG per mouse for analysis at day 7. Alternatively, B1-8^hi^ IgH knock-in mice were bred with CD45.1 mice to generate B1-8^hi^WT (IgMa, CD45.1/CD45.2) mice. 5 × 10^6^ CTV-labeled naive spleen B cells mixed in 1:1 ratio from B1-8^hi^AID^R112H^ mice (IgMa, CD45.2) and B1-8^hi^WT mice (IgMa, CD45.1/CD45.2) were injected i.v. into WT recipient mice (IgMb, CD45.1), followed by NP-CGG immunization for 7 days. The S1pr2-ERT2cre mice were generously provided by T. Kurosaki, Osaka University, Osaka, Japan (Shinnakasu et al., 2016), and Rosa26-tdTomato mice (Ai14; Rosa-CAG-LSL-tdTomato-WPRE, Stock No. 007914) were purchased from The Jackson Laboratory (Termote et al., 2025).

### B cell isolation and iGB cell culture

Mouse splenic B cells were enriched by the negative B cell selection kit from StemCell Technology. B cells were cultured at 1.6 × 10^4^ cell/ml on top of 80 Gy-irradiated 40LB cells (Nojima et al., 2011). Cell culture was supplemented with 1–2 ng/ml of mouse IL-4 (PeproTech). On day 4, 2–4 × 10^3^ cells from this culture were replated on freshly irradiated 40LB cells with 10–20 ng/ml of mouse IL-21 (PeproTech) for another 4–6 days to induce PC differentiation. 10 ng/ml of topoisomerase inhibitor etoposide was added on day 4 for 48 or 96 h 500 ng/ml 5-FU or 50 ng/ml PEM was added on day 4 for 24 h. To induce T-independent activation of B cells in vitro, 4 × 10^5^ cells/ml of isolated B cells were cultured with complete RPMI 1640 with 10 µg/ml LPS for up to 4 days.

### EdU and BrdU labeling

iGB cultures were pulsed with 20 uM of EdU for 15 min and washed away, followed by labeling with BrdU for 5 min, or 1, 2, or 4 h before harvesting the culture. Detection of EdU was performed using the Click-It EdU kit (Thermo Fisher Scientific). BrdU was detected using monoclonal anti-BrdU antibody.

### Immunization and flow cytometry

WT, AID^R112H^, and WT:AID^R112H^ bone marrow chimeric mice were immunized with SRBCs via i.p. injections. Seven days after immunization, single-cell suspensions were prepared from spleen, lymph nodes, and bone marrow, and labeled with fluorescence-conjugated antibodies including B220, CD43, CD24, CD23, CD21, IgM, IgD, IgG1, CD93, CD138, GL7, CD95, CXCR4, CXCR5, CD83, CD86, CD73, PD-L2, CD80, Blimp1, PAX5, IRF4, BCL6, FOXO1, CD45.1, CD45.2, IgMa, IgMb, anti-BrdU. Data were obtained by LSRFortessa flow cytometry (BD) and analyzed with FlowJo software (TreeStar).

### Western blot and immunoprecipitation

iGB cells were washed with cold PBS and then lysed in radioimmunoprecipitation assay buffer (150 mM NaCl, 1.0% IGEPAL, 0.5% sodium deoxycholate, 0.1% SDS, 50 mM Tris, pH 8.0) containing protease and phosphatase inhibitors for 1 h on ice. For immunoprecipitation, 3 μg of antibody was added to the cell lysate from 5 × 10^6^ cells per antibody, and then rotated at 4° overnight. Immune complexes were pulled down using 10 µl Protein A/G magnetic beads (Cat. #88803; Thermo Fisher Scientific) for 3 h at 4°. The protein was eluted with 60 μl of 2× SDS by boiling for 5 min at 95°. Anti-TET2 (Cat. #ab124297; Abcam), anti-DNMT1 (Cat. #39204; Active Motif), anti-AID (Cat. #39885; Active Motif), anti-AID (eBioscience, Cat. #14-5959-82; Thermo Fisher Scientific), anti-H3K4me3, and anti-IgG (present in CST #91931 kit) antibodies were used in the experiments.

### CUT&RUN

5 × 10^5^ cells were processed with CUT&RUN Assay Kit (Cat. #91931; Cell Signaling Technology) according to the manufacturer’s instructions. 4 µg of anti-AID (Cat. #39885; Active Motif), anti-TET2 (Cat. #ab124297; Abcam), anti-DNMT1 (Cat. #39204; Active Motif), anti-H3K4me3, and anti-IgG (present in CST #91931 kit) antibodies were used in the experiments. CUT&RUN DNA products were purified using QIAquick PCR Purification Kit (Cat. #28106) and quantified with real-time PCR using SsoAdvanced SYBR Green Supermix (Cat. #1725274; Bio-Rad). Primer sequences are shown in Table S1.

### CUT&RUN-sequencing analysis

Paired-end (150-bp) sequencing was performed by Novogene, using the Illumina NovaSeq 6000 platform. Raw reads were quality-filtered with Cutadapt 4.9, and mapped to both *Mus musculus* (mm39) and *Saccharomyces cerevisiae* (sacCer3, spike-in) genomes with Bowtie2 2.5.4 (Langmead and Salzberg, 2012) using the following options: end-to-end --very-sensitive --no-mixed --no-discordant. After normalization with the spike-in raw counts and removal of off-target sequences (Nordin et al., 2023), normalized bedGraph files were generated with bedtools 2.31.0 and converted to bigWig files using UCSC’s bedGraphToBigWig tool (Quinlan and Hall, 2010; Kent et al., 2010). Visualization was done in the UCSC Genome Browser, and TSS-centered heatmaps and profiles were generated with deepTools 3.5.5 (Ramírez et al., 2016).

### DNA methylation analysis by bisulfite treatment

Inducible GC B cells from day 7 were subjected to CpG methylation analysis using EZ DNA Methylation-Direct Kit (Cat. #D5021; Zymo Research). In brief, 1 × 10^4^ iGB cells were lysed for 20 min at 50° with M-digestion buffer containing proteinase K. Cell lysates containing DNA were directly subjected to bisulfite conversion for 3.5 h. Reaction was stopped using desulfonation buffer, and then, DNA was purified with a Zymo-Spin column. The modified DNA was amplified by PCR. PCR products were cloned into a pCR4-TOPO TA vector and sequenced (Cat. #K457501; Thermo Fisher Scientific). Primer sequences are shown in Table S1.

### Immunofluorescence staining

Spleens from unimmunized and SRBC-immunized mice were imbedded with Tissue-Tek OCT compound (Sakura) for immunofluorescence staining. 8-mm-thick tissue sections were cut from the imbedded spleen using a cryostat microtome. Sections were fixed in ice-cold acetone for 10 min and blocked with 5% fetal calf serum for 1 h. The sections were incubated with primary antibodies overnight at 4°C, washed with PBS, and then incubated with secondary antibodies for 45 min. Fluorescence images were captured using Leica DM IRBE confocal microscope that has one argon laser and two HeNe lasers. HC PL APO lens at 20×/0.70 CS was used at room temperature. Images were processed with ImageJ software.

### Genotyping

Exon 2 from the *aicda* gene was amplified by Taq polymerase with the following primers: 5′-TCT​GGC​TGC​CAC​GTG​GAA​TTG​T-3′ and 5′-TGA​TCC​CGA​TCT​GGA​CCC​CAG​C-3′. PCR program steps were as follows: 95°C, 2 min, 30 cycles of 95°C for 30 s, 63°C for 30 s, and 72°C for 30 s, and a last step of 72°C for 10 min. The PCR product was digested with 1U BssHII at 37°C overnight. PCR products from WT alleles would be cut into two fragments (175- and 84-bp), whereas the AID^R112H^ product would be resistant to the digestion.

### RNA-sequencing analysis and GSEA

Total RNA was purified from sorted cells using RNeasy kit #74104 (Qiagen). Each RNA sample was treated with an RNase-free DNase kit #79254 (Qiagen) during the RNA isolation procedure. The concentration of purified total RNA was measured by NanoDrop 2000 (Thermo Fisher Scientific). RNA sequencing was performed using the Illumina HiSeq PE150 platform at Novogene, Hong Kong, China. Reads were aligned to the reference genome of mouse (*M. musculus*) assembly December 2011 (GRCm38/mm10).

The analysis was conducted with R 4.2.3. Briefly, raw gene counts were used as inputs of DESeq2 package (1.38.3) for differential gene expression (DGE) analysis. Prefilter conditions include total gene count <10 and immunoglobulin variable genes. Marker genes from each condition were identified by ranking the product of log fold change and log-transformed adjusted P value times −1. Identified marker genes were visualized using ComplexHeatmap package (2.14.0) with z-score of variance-stabilizing–transformed gene counts. The GSEA was performed with clusterProfiler package (4.4.4) with full-ranked DGE result and published marker genes for different stages of B cells (Gómez-Escolar et al., 2022) as input files of GSEA command. Pathway enrichment analysis was done in two steps. First, the enriched gene ontology terms were identified for each ranked gene list with the gseGO() function. Following that, enriched terms were visualized using Cytoscape (3.9.1).

### Structural modeling analysis of AID

The 3D models of WT and mutated AID were built with RoseTTAFold (Baek et al., 2021). PyMOL 3.1 was used for visualization of the structures (https://pymol.org/). The RMSD was used to evaluate structural divergence among the predicted models. The mutated AID predicted models were aligned with the WT AID, and the RMSD was measured with PyMOL 3.1. To predict mutation effects on protein stability, we calculated the ΔΔG for 26 single point mutations in AID using DDMut web server (https://biosig.lab.uq.edu.au/ddmut/) (Zhou et al., 2023). The surface electrostatic potential was evaluated using the Adaptive Poisson–Boltzmann Solver PyMOL plugin (Jurrus et al., 2018). To further elucidate the impact of the R112H mutation, the interatomic interactions of WT and AID^R112H^ were evaluated with Arpeggio (Jubb et al., 2017). Figures were prepared using PyMOL 3.1. The ssDNA (5′- AGT​AGT​TAT​CAG​CTA​TGC​TCA​GTG-3′) was built using the program 3DNA (Lu and Olson, 2003), and the AID models were generated using RoseTTAFold. The protein–DNA complex was formed by docking using HADDOCK software with default parameters (van Dijk et al., 2013). The docked complex was selected from the top-ranked clustered structures.

### Statistics

Statistical analysis was performed as indicated in the figure legends. Statistics was performed using GraphPad Prism version 8. P ≤ 0.05, significant; P > 0.05, nonsignificant.

### Online supplemental material

Fig. S1 includes flow cytometry data on B cell differentiation in vivo from regular mice or bone marrow chimeric mice, immunofluorescence images of spleen sections (E), and heatmap from bulk RNA sequencing of GC B cells and GL7^lo/−^ B cells. Fig. S2 includes flow cytometry data on GC fate mapping, expression of BCL6 and CXCR5, in vitro B cell proliferation, and IRF4 ectopic expression; and GO analysis of bulk RNA sequencing of GC B cells and GL7^lo/−^ B cells. Fig. S3 includes flow cytometry data on high-affinity B cell differentiation in vivo, Ugi-ectopic expression and cell proliferation, and Ig class switching of TET2 KO B cells. DNA methylation data of naive B cells are included. Fig. S4 includes full images of all co-immunoprecipitation data and western blot data. Fig. S4 displays the peak profiles of selected genes from CUT&RUN-sequencing data. Table S1 shows primer sequences.

## Supplementary Material

Table S1 shows primer sequences.

SourceData F8 is the source file for Fig. 8.

SourceData FS4 is the source file for Fig. S4.

## Data Availability

The data for transcriptomics and CUT&RUN sequencing are stored at European Nucleotide Archive (https://www.ebi.ac.uk/ena) with the accession number ERP170279. All the data described in the text are available in main figures and supplementary figures. Additional raw data are available upon reasonable request.

## References

[bib1] Álvarez-Prado, Á.F., P. Pérez-Durán, A. Pérez-García, A. Benguria, C. Torroja, V.G. de Yébenes, and A.R. Ramiro. 2018. A broad atlas of somatic hypermutation allows prediction of activation-induced deaminase targets. J. Exp. Med. 215:761–771. 10.1084/jem.20171738 29374026 PMC5839764

[bib2] Baek, M., F. DiMaio, I. Anishchenko, J. Dauparas, S. Ovchinnikov, G.R. Lee, J. Wang, Q. Cong, L.N. Kinch, R. Dustin Schaeffer, . 2021. Accurate prediction of protein structures and interactions using a three-track neural network. Science. 373:871–876. 10.1126/science.abj8754 34282049 PMC7612213

[bib3] Barwick, B.G., C.D. Scharer, A.P.R. Bally, and J.M. Boss. 2016. Plasma cell differentiation is coupled to division-dependent DNA hypomethylation and gene regulation. Nat. Immunol. 17:1216–1225. 10.1038/ni.3519 27500631 PMC5157049

[bib5] Carpenter, M.A., E. Rajagurubandara, P. Wijesinghe, and A.S. Bhagwat. 2010. Determinants of sequence-specificity within human AID and APOBEC3G. DNA Repair. 9:579–587. 10.1016/J.DNAREP.2010.02.010 20338830 PMC2878719

[bib6] Casellas, R., U. Basu, W.T. Yewdell, J. Chaudhuri, D.F. Robbiani, and J.M. Di Noia. 2016. Mutations, kataegis and translocations in B cells: Understanding AID promiscuous activity. Nat. Rev. Immunol. 16:164–176. 10.1038/nri.2016.2 26898111 PMC4871114

[bib7] Català-Moll, F., A.G. Ferreté-Bonastre, T. Li, D. Weichenhan, P. Lutsik, L. Ciudad, Á.F. Álvarez-Prado, J. Rodríguez-Ubreva, C. Klemann, C. Speckmann, . 2021. Activation-induced deaminase is critical for the establishment of DNA methylation patterns prior to the germinal center reaction. Nucleic Acids Res. 49:5057–5073. 10.1093/nar/gkab322 33950194 PMC8136777

[bib8] Chiarle, R., Y. Zhang, R.L. Frock, S.M. Lewis, B. Molinie, Y.-J. Ho, D.R. Myers, V.W. Choi, M. Compagno, D.J. Malkin, . 2011. Genome-wide translocation sequencing reveals mechanisms of chromosome breaks and rearrangements in B cells. Cell. 147:107–119. 10.1016/j.cell.2011.07.049 21962511 PMC3186939

[bib10] Dahlberg, C.I.M., M. He, T. Visnes, M.L. Torres, E.M. Cortizas, R.E. Verdun, L.S. Westerberg, E. Severinson, and L. Ström. 2014. A novel mouse model for the hyper-IgM syndrome: A spontaneous activation-induced cytidine deaminase mutation leading to complete loss of Ig class switching and reduced somatic hypermutation. J. Immunol. 193:4732–4738. 10.4049/jimmunol.1401242 25252954 PMC4201989

[bib11] Daniel, J.A., and A. Nussenzweig. 2013. The AID-induced DNA damage response in chromatin. Mol. Cell. 50:309–321. 10.1016/j.molcel.2013.04.017 23664375 PMC3658174

[bib12] Di Noia, J., and M.S. Neuberger. 2002. Altering the pathway of immunoglobulin hypermutation by inhibiting uracil-DNA glycosylase. Nature. 419:43–48. 10.1038/nature00981 12214226

[bib13] Di Noia, J.M., and M.S. Neuberger. 2007. Molecular mechanisms of antibody somatic hypermutation. Annu. Rev. Biochem. 76:1–22. 10.1146/annurev.biochem.76.061705.090740 17328676

[bib14] Dominguez, P.M., H. Ghamlouch, W. Rosikiewicz, P. Kumar, W. Béguelin, L. Fontán, M.A. Rivas, P. Pawlikowska, M. Armand, E. Mouly, . 2018. TET2 deficiency causes germinal center hyperplasia, impairs plasma cell differentiation, and promotes b-cell lymphomagenesis. Cancer Discov. 8:1633–1653. 10.1158/2159-8290.CD-18-0657 PMC6279514 30274972

[bib15] Dominguez, P.M., M. Teater, N. Chambwe, M. Kormaksson, D. Redmond, J. Ishii, B. Vuong, J. Chaudhuri, A. Melnick, A. Vasanthakumar, . 2015. DNA methylation dynamics of germinal center B cells are mediated by AID. Cell Rep. 12:2086–2098. 10.1016/j.celrep.2015.08.036 26365193 PMC4591215

[bib16] Dominguez-Sola, D., J. Kung, A.B. Holmes, V.A. Wells, T. Mo, K. Basso, and R. Dalla-Favera. 2015. The FOXO1 transcription factor instructs the germinal center dark zone program. Immunity. 43:1064–1074. 10.1016/j.immuni.2015.10.015 26620759

[bib17] Fujii, K., S. Tanaka, T. Hasegawa, M. Narazaki, A. Kumanogoh, H. Koseki, T. Kurosaki, and W. Ise. 2020. Tet DNA demethylase is required for plasma cell differentiation by controlling expression levels of IRF4. Int. Immunol. 32:683–690. 10.1093/INTIMM/DXAA042 32583857

[bib18] Gitlin, A.D., Z. Shulman, and M.C. Nussenzweig. 2014. Clonal selection in the germinal centre by regulated proliferation and hypermutation. Nature. 509:637–640. 10.1038/nature13300 24805232 PMC4271732

[bib19] Glaros, V., R. Rauschmeier, A.V. Artemov, A. Reinhardt, S. Ols, A. Emmanouilidi, C. Gustafsson, Y. You, C. Mirabello, Å.K. Björklund, . 2021. Limited access to antigen drives generation of early B cell memory while restraining the plasmablast response. Immunity. 54:2005–2023.e10. 10.1016/j.immuni.2021.08.017 34525339 PMC7612941

[bib20] Gómez‐Escolar, C., A. Serrano‐Navarro, A. Benguria, A. Dopazo, F. Sánchez‐Cabo, and A.R. Ramiro. 2022. Single cell clonal analysis identifies an AID-dependent pathway of plasma cell differentiation. EMBO Rep. 23:e55000. 10.15252/embr.202255000 36205653 PMC9724673

[bib22] Jubb, H.C., A.P. Higueruelo, B. Ochoa-Montaño, W.R. Pitt, D.B. Ascher, and T.L. Blundell. 2017. Arpeggio: A web server for calculating and visualising interatomic interactions in protein structures. J. Mol. Biol. 429:365–371. 10.1016/j.jmb.2016.12.004 27964945 PMC5282402

[bib23] Jurrus, E., D. Engel, K. Star, K. Monson, J. Brandi, L.E. Felberg, D.H. Brookes, L. Wilson, J. Chen, K. Liles, . 2018. Improvements to the APBS biomolecular solvation software suite. Protein Sci. 27:112–128. 10.1002/pro.3280 28836357 PMC5734301

[bib24] Kent, W.J., A.S. Zweig, G. Barber, A.S. Hinrichs, and D. Karolchik. 2010. BigWig and BigBed: Enabling browsing of large distributed datasets. Bioinformatics. 26:2204–2207. 10.1093/bioinformatics/btq351 20639541 PMC2922891

[bib25] King, J.J., C.A. Manuel, C.V. Barrett, S. Raber, H. Lucas, P. Sutter, and M. Larijani. 2015. Catalytic pocket inaccessibility of activation-induced cytidine deaminase is a safeguard against excessive mutagenic activity. Structure. 23:615–627. 10.1016/j.str.2015.01.016 25728927

[bib26] Klein, U., and R. Dalla-Favera. 2008. Germinal centres: Role in B-cell physiology and malignancy. Nat. Rev. Immunol. 8:22–33. 10.1038/nri2217 18097447

[bib27] Kodgire, P., P. Mukkawar, S. Ratnam, T.E. Martin, and U. Storb. 2013. Changes in RNA polymerase II progression influence somatic hypermutation of Ig-related genes by AID. J. Exp. Med. 210:1481–1492. 10.1084/jem.20121523 23752228 PMC3698518

[bib28] Kohli, R.M., S.R. Abrams, K.S. Gajula, R.W. Maul, P.J. Gearhart, and J.T. Stivers. 2009. A portable hot spot recognition loop transfers sequence preferences from APOBEC family members to activation-induced cytidine deaminase. J. Biol. Chem. 284:22898–22904. 10.1074/jbc.M109.025536 19561087 PMC2755697

[bib30] Kuraoka, M., T.M. Holl, D. Liao, M. Womble, D.W. Cain, A.E. Reynolds, and G. Kelsoe. 2011. Activation-induced cytidine deaminase mediates central tolerance in B cells. Proc. Natl. Acad. Sci. USA. 108:11560–11565. 10.1073/pnas.1102571108/-/DCSupplemental 21700885 PMC3136303

[bib32] Langmead, B., and S.L. Salzberg. 2012. Fast gapped-read alignment with Bowtie 2. Nat. Methods 9:357–359. 10.1038/nmeth.1923 22388286 PMC3322381

[bib33] Li, X., A. Gadzinsky, L. Gong, H. Tong, V. Calderon, Y. Li, D. Kitamura, U. Klein, W.Y. Langdon, F. Hou, . 2018. Cbl ubiquitin ligases control B cell exit from the germinal-center reaction. Immunity. 48:530–541.e6. 10.1016/j.immuni.2018.03.006 29562201

[bib34] Lio, C.-W.J., V. Shukla, D. Samaniego-Castruita, E. González-Avalos, A. Chakraborty, X. Yue, D.G. Schatz, F. Ay, and A. Rao. 2019. TET enzymes augment activation-induced deaminase (AID) expression via 5-hydroxymethylcytosine modifications at the Aicda superenhancer. Sci. Immunol. 4:eaau7523. 10.1126/sciimmunol.aau7523 31028100 PMC6599614

[bib36] Liu, M., J.L. Duke, D.J. Richter, C.G. Vinuesa, C.C. Goodnow, S.H. Kleinstein, and D.G. Schatz. 2008. Two levels of protection for the B cell genome during somatic hypermutation. Nature. 451:841–845. 10.1038/nature06547 18273020

[bib37] Longley, D.B., D.P. Harkin, and P.G. Johnston. 2003. 5-Fluorouracil: Mechanisms of action and clinical strategies. Nat. Rev. Cancer 3:330–338. 10.1038/nrc1074 12724731

[bib38] Lu, X.-J., and W.K. Olson. 2003. 3DNA: A software package for the analysis, rebuilding and visualization of three-dimensional nucleic acid structures. Nucleic Acids Res. 31:5108–5121. 10.1093/nar/gkg680 12930962 PMC212791

[bib40] Maul, R.W., Z. Cao, L. Venkataraman, C.A. Giorgetti, J.L. Press, Y. Denizot, H. Du, R. Sen, and P.J. Gearhart. 2014. Spt5 accumulation at variable genes distinguishes somatic hypermutation in germinal center B cells from ex vivo-activated cells. J. Exp. Med. 211:2297–2306. 10.1084/jem.20131512 25288395 PMC4203944

[bib41] Mayer, C.T., A. Gazumyan, E.E. Kara, A.D. Gitlin, J. Golijanin, C. Viant, J. Pai, T.Y. Oliveira, Q. Wang, A. Escolano, . 2017. The microanatomic segregation of selection by apoptosis in the germinal center. Science. 358:eaao2602. 10.1126/science.aao2602 28935768 PMC5957278

[bib42] Meng, F.-L., Z. Du, A. Federation, J. Hu, Q. Wang, K.R. Kieffer-Kwon, R.M. Meyers, C. Amor, C.R. Wasserman, D. Neuberg, . 2014. Convergent transcription at intragenic super-enhancers targets AID-initiated genomic instability. Cell. 159:1538–1548. 10.1016/j.cell.2014.11.014 25483776 PMC4322776

[bib43] Methot, S.P., and J.M. Di Noia. 2017. Molecular mechanisms of somatic hypermutation and class switch recombination. Adv. Immunol. 133:37–87. 10.1016/bs.ai.2016.11.002 28215280

[bib44] Meyers, G., Y.S. Ng, J.M. Bannock, A. Lavoie, J.E. Walter, L.D. Notarangelo, S.S. Kilic, G. Aksu, M. Debré, F. Rieux-Laucat, . 2011. Activation-induced cytidine deaminase (AID) is required for B-cell tolerance in humans. Proc. Natl. Acad. Sci. USA. 108:11554–11559. 10.1073/pnas.1102600108 21700883 PMC3136251

[bib45] Morgan, H.D., W. Dean, H.a Coker, W. Reik, and S.K. Petersen-Mahrt. 2004. Activation-induced cytidine deaminase deaminates 5-methylcytosine in DNA and is expressed in pluripotent tissues: Implications for epigenetic reprogramming. J. Biol. Chem. 279:52353–52360. 10.1074/jbc.M407695200 15448152

[bib46] Muramatsu, M., K. Kinoshita, S. Fagarasan, S. Yamada, Y. Shinkai, and T. Honjo. 2000. Class switch recombination and hypermutation require activation-induced cytidine deaminase (AID), a potential RNA editing enzyme. Cell. 102:553–563. 10.1016/s0092-8674(00)00078-7 11007474

[bib47] Muramatsu, M., V.S. Sankaranand, S. Anant, M. Sugai, K. Kinoshita, N.O. Davidson, and T. Honjo. 1999. Specific expression of activation-induced cytidine deaminase (AID), a novel member of the RNA-editing deaminase family in germinal center B cells. J. Biol. Chem. 274:18470–18476. 10.1074/jbc.274.26.18470 10373455

[bib49] Nambu, Y., M. Sugai, H. Gonda, C.-G. Lee, T. Katakai, Y. Agata, Y. Yokota, and A. Shimizu. 2003. Transcription-coupled events associating with immunoglobulin switch region chromatin. Science. 302:2137–2140. 10.1126/science.1092481 14684824

[bib50] Nera, K.-P., P. Kohonen, E. Narvi, A. Peippo, L. Mustonen, P. Terho, K. Koskela, J.M. Buerstedde, and O. Lassila. 2006. Loss of Pax5 promotes plasma cell differentiation. Immunity. 24:283–293. 10.1016/j.immuni.2006.02.003 16546097

[bib51] Nojima, T., K. Haniuda, T. Moutai, M. Matsudaira, S. Mizokawa, I. Shiratori, T. Azuma, and D. Kitamura. 2011. In-vitro derived germinal centre B cells differentially generate memory B or plasma cells in vivo. Nat. Commun. 2:465. 10.1038/ncomms1475 21897376

[bib52] Nordin, A., G. Zambanini, P. Pagella, and C. Cantù. 2023. The CUT&RUN suspect list of problematic regions of the genome. Genome Biol. 24:185. 10.1186/s13059-023-03027-3 37563719 PMC10416431

[bib53] Nutt, S.L., P.D. Hodgkin, D.M. Tarlinton, and L.M. Corcoran. 2015. The generation of antibody-secreting plasma cells. Nat. Rev. Immunol. 15:160–171. 10.1038/nri3795 25698678

[bib55] Omori, S.A., M.H. Cato, A. Anzelon-Mills, K.D. Puri, M. Shapiro-Shelef, K. Calame, and R.C. Rickert. 2006. Regulation of class-switch recombination and plasma cell differentiation by phosphatidylinositol 3-kinase signaling. Immunity. 25:545–557. 10.1016/j.immuni.2006.08.015 17000121

[bib56] Papin, A., E. Cesarman, and A. Melnick. 2022. 3D chromosomal architecture in germinal center B cells and its alterations in lymphomagenesis. Curr. Opin. Genet. Dev. 74:101915. 10.1016/j.gde.2022.101915 35550952 PMC9254163

[bib57] Pastor, W.A., L. Aravind, and A. Rao. 2013. TETonic shift: Biological roles of TET proteins in DNA demethylation and transcription. Nat. Rev. Mol. Cell Biol. 14:341–356. 10.1038/nrm3589 23698584 PMC3804139

[bib58] Pavri, R., A. Gazumyan, M. Jankovic, M. Di Virgilio, I. Klein, C. Ansarah-Sobrinho, W. Resch, A. Yamane, B. Reina San-Martin, V. Barreto, . 2010. Activation-induced cytidine deaminase targets DNA at sites of RNA polymerase II stalling by interaction with Spt5. Cell. 143:122–133. 10.1016/j.cell.2010.09.017 20887897 PMC2993080

[bib59] Pefanis, E., J. Wang, G. Rothschild, J. Lim, J. Chao, R. Rabadan, A.N. Economides, and U. Basu. 2014. Noncoding RNA transcription targets AID to divergently transcribed loci in B cells. Nature. 514:389–393. 10.1038/nature13580 25119026 PMC4372240

[bib60] Phan, R.T., M. Saito, Y. Kitagawa, A.R. Means, and R. Dalla-Favera. 2007. Genotoxic stress regulates expression of the proto-oncogene Bcl6 in germinal center B cells. Nat. Immunol. 8:1132–1139. 10.1038/ni1508 17828269

[bib61] Prochnow, C., R. Bransteitter, M.G. Klein, M.F. Goodman, and X.S. Chen. 2007. The APOBEC-2 crystal structure and functional implications for the deaminase AID. Nature. 445:447–451. 10.1038/nature05492 17187054

[bib62] Qian, J., Q. Wang, M. Dose, N. Pruett, K.R. Kieffer-Kwon, W. Resch, G. Liang, Z. Tang, E. Mathé, C. Benner, . 2014. B cell super-enhancers and regulatory clusters recruit AID tumorigenic activity. Cell. 159:1524–1537. 10.1016/j.cell.2014.11.013 25483777 PMC4272762

[bib63] Qiao, Q., L. Wang, F.L. Meng, J.K. Hwang, F.W. Alt, and H. Wu. 2017. AID recognizes structured DNA for class switch recombination. Mol. Cell. 67:361–373.e4. 10.1016/j.molcel.2017.06.034 28757211 PMC5771415

[bib64] Quinlan, A.R., and I.M. Hall. 2010. BEDTools: A flexible suite of utilities for comparing genomic features. Bioinformatics. 26:841–842. 10.1093/bioinformatics/btq033 20110278 PMC2832824

[bib65] Rada, C., J.M. Di Noia, and M.S. Neuberger. 2004. Mismatch recognition and uracil excision provide complementary paths to both Ig switching and the A/T-focused phase of somatic mutation. Mol. Cell. 16:163–171. 10.1016/j.molcel.2004.10.011 15494304

[bib66] Ramírez, F., D.P. Ryan, B. Grüning, V. Bhardwaj, F. Kilpert, A.S. Richter, S. Heyne, F. Dündar, and T. Manke. 2016. deepTools2: a next generation web server for deep-sequencing data analysis. Nucleic Acids Res. 44:W160–W165. 10.1093/NAR/GKW257 27079975 PMC4987876

[bib67] Ramiro, A.R., and V.M. Barreto. 2015. Activation-induced cytidine deaminase and active DNA demethylation. Trends Biochem. Sci. 40:172–181. 10.1016/j.tibs.2015.01.006 25661247

[bib68] Revy, P., T. Muto, Y. Levy, F. Geissmann, A. Plebani, O. Sanal, N. Catalan, M. Forveille, R. Dufourcq-Labelouse, A. Gennery, . 2000. Activation-induced cytidine deaminase (AID) deficiency causes the autosomal recessive form of the Hyper-IgM syndrome (HIGM2). Cell. 102:565–575. 10.1016/s0092-8674(00)00079-9 11007475

[bib69] Rosikiewicz, W., X. Chen, P.M. Dominguez, H. Ghamlouch, S. Aoufouchi, O.A. Bernard, A. Melnick, and S. Li. 2020. TET2 deficiency reprograms the germinal center B cell epigenome and silences genes linked to lymphomagenesis. Sci. Adv. 6:eaay5872. 10.1126/sciadv.aay5872 32596441 PMC7299612

[bib71] Sander, S., V.T. Chu, T. Yasuda, A. Franklin, R. Graf, D.P. Calado, S. Li, K. Imami, M. Selbach, M. Di Virgilio, . 2015. PI3 kinase and FOXO1 transcription factor activity differentially control B cells in the germinal center light and dark zones. Immunity. 43:1075–1086. 10.1016/j.immuni.2015.10.021 26620760

[bib72] Schoeler, K., A. Aufschnaiter, S. Messner, E. Derudder, S. Herzog, A. Villunger, K. Rajewsky, and V. Labi. 2019. TET enzymes control antibody production and shape the mutational landscape in germinal centre B cells. FEBS J. 286:3566–3581. 10.1111/febs.14934 31120187 PMC6851767

[bib74] Shaknovich, R., L. Cerchietti, L. Tsikitas, M. Kormaksson, S. De, M.E. Figueroa, G. Ballon, S.N. Yang, N. Weinhold, M. Reimers, . 2011. DNA methyltransferase 1 and DNA methylation patterning contribute to germinal center B-cell differentiation. Blood. 118:3559–3569. 10.1182/blood-2011-06-357996 21828137 PMC3186332

[bib75] Shapiro-Shelef, M., and K. Calame. 2005. Regulation of plasma-cell development. Nat. Rev. Immunol. 5:230–242. 10.1038/nri1572 . 15738953

[bib76] Shih, T.-A.Y., M. Roederer, and M.C. Nussenzweig. 2002. Role of antigen receptor affinity in T cell-independent antibody responses in vivo. Nat. Immunol. 3:399–406. 10.1038/ni776 11896394

[bib77] Shinnakasu, R., T. Inoue, K. Kometani, S. Moriyama, Y. Adachi, M. Nakayama, Y. Takahashi, H. Fukuyama, T. Okada, and T. Kurosaki. 2016. Regulated selection of germinal-center cells into the memory B cell compartment. Nat. Immunol. 17:861–869. 10.1038/ni.3460 27158841

[bib78] Shlomchik, M.J., and F. Weisel. 2012. Germinal center selection and the development of memory B and plasma cells. Immunol. Rev. 247:52–63. 10.1111/j.1600-065X.2012.01124.x 22500831

[bib81] Stavnezer, J., J.E.J. Guikema, and C.E. Schrader. 2008. Mechanism and regulation of class switch recombination. Annu. Rev. Immunol. 26:261–292. 10.1146/annurev.immunol.26.021607.090248 18370922 PMC2707252

[bib82] Stewart, I., D. Radtke, B. Phillips, S.J. McGowan, and O. Bannard. 2018. Germinal center B cells replace their antigen receptors in dark zones and fail light zone entry when immunoglobulin gene mutations are damaging. Immunity. 49:477–489.e7. 10.1016/j.immuni.2018.08.025 30231983 PMC6162340

[bib83] Termote, M., R.C. Marques, E. Hyllner, M.V. Guryleva, M. Henskens, A. Brutscher, I.J.L. Baken, X.C. Dopico, A.D. Gasull, B. Murrell, . 2025. Antigen affinity and site of immunization dictate B cell recall responses. Cell Rep. 44:115221. 10.1016/j.celrep.2024.115221 39817910

[bib84] Tunyaplin, C., A.L. Shaffer, C.D. Angelin-Duclos, X. Yu, L.M. Staudt, and K.L. Calame. 2004. Direct repression of prdm1 by Bcl-6 inhibits Plasmacytic differentiation. J. Immunol. 173:1158–1165. 10.4049/jimmunol.173.2.1158 15240705

[bib85] van Dijk, M., K.M. Visscher, P.L. Kastritis, and A.M.J.J. Bonvin. 2013. Solvated protein-DNA docking using HADDOCK. J. Biomol. NMR. 56:51–63. 10.1007/s10858-013-9734-x 23625455

[bib86] Venturutti, L., and A.M. Melnick. 2021. The role of epigenetic mechanisms in B cell lymphoma pathogenesis. Annu. Rev. Cancer Biol. 5:311–330. 10.1146/annurev-cancerbio-060820-125304

[bib87] Victora, G.D., and M.C. Nussenzweig. 2012. Germinal centers. Annu. Rev. Immunol. 30:429–457. 10.1146/annurev-immunol-020711-075032 22224772

[bib88] Victora, G.D., T.A. Schwickert, D.R. Fooksman, A.O. Kamphorst, M. Meyer-Hermann, M.L. Dustin, and M.C. Nussenzweig. 2010. Germinal center dynamics revealed by multiphoton microscopy with a photoactivatable fluorescent reporter. Cell. 143:592–605. 10.1016/j.cell.2010.10.032 21074050 PMC3035939

[bib90] Wang, M., C. Rada, and M.S. Neuberger. 2010. Altering the spectrum of immunoglobulin V gene somatic hypermutation by modifying the active site of AID. J. Exp. Med. 207:141–153. 10.1084/JEM.20092238 20048284 PMC2812546

[bib91] Wang, X., M. Fan, S. Kalis, L. Wei, and M.D. Scharff. 2014. A source of the single-stranded DNA substrate for activation-induced deaminase during somatic hypermutation. Nat. Commun. 5:4137. 10.1038/ncomms5137 24923561 PMC4154566

[bib92] Wu, S., Y. Yin, and X. Wang. 2022. The epigenetic regulation of the germinal center response. Biochim. Biophys. Acta Gene Regul. Mech. 1865:194828. 10.1016/j.bbagrm.2022.194828 35643396

[bib93] Wu, X., and Y. Zhang. 2017. TET-mediated active DNA demethylation: Mechanism, function and beyond. Nat. Rev. Genet. 18:517–534. 10.1038/nrg.2017.33 28555658

[bib94] Yamane, A., W. Resch, N. Kuo, S. Kuchen, Z. Li, H. Sun, D.F. Robbiani, K. McBride, M.C. Nussenzweig, and R. Casellas. 2011. Deep-sequencing identification of the genomic targets of the cytidine deaminase AID and its cofactor RPA in B lymphocytes. Nat. Immunol. 12:62–69. 10.1038/ni.1964 21113164 PMC3005028

[bib95] Yan, C.T., C. Boboila, E.K. Souza, S. Franco, T.R. Hickernell, M. Murphy, S. Gumaste, M. Geyer, A.A. Zarrin, J.P. Manis, . 2007. IgH class switching and translocations use a robust non-classical end-joining pathway. Nature. 449:478–482. 10.1038/nature06020 17713479

[bib96] Zaheen, A., B. Boulianne, J.Y. Parsa, S. Ramachandran, J.L. Gommerman, and A. Martin. 2009. AID constrains germinal center size by rendering B cells susceptible to apoptosis. Blood. 114:547–554. 10.1182/blood-2009-03-211763 19478044

[bib98] Zhang, Y., M. Meyer-Hermann, L. a George, M.T. Figge, M. Khan, M. Goodall, S.P. Young, A. Reynolds, F. Falciani, A. Waisman, . 2013. Germinal center B cells govern their own fate via antibody feedback. J. Exp. Med. 210:457–464. 10.1084/jem.20120150 23420879 PMC3600904

[bib99] Zhou, Y., Q. Pan, D.E.V. Pires, C.H.M. Rodrigues, and D.B. Ascher. 2023. DDMut: Predicting effects of mutations on protein stability using deep learning. Nucleic Acids Res. 51:W122–W128. 10.1093/nar/gkad472 37283042 PMC10320186

