# Supplementary material for: AID and TET2 cooperate to demethylate Irf4 for plasma cell fate in germinal center B cells
Source: J Exp Med. 2026 Apr 27;223(6):e20260096. doi: 10.1084/jem.20260096 (PMC13116153; doi:10.1084/jem.20260096)
Supplement: Table S1 — shows primer sequences. [file jem_20260096_tables1.docx]

**Table S1.** Primer sequences

| Cut&Run primers | | |
| --- | --- | --- |
| IRF4-P3 | C&R_IRF4-P3F | 5’-GGTGAAAGAACGACATGGCATC-3’ |
|  | C&R_IRF4-P3R | 5’-TTCCATCCTGGGTTCTGGACT-3’ |
| IRF4-P4 | C&R_IRF4-P4F | 5’-CCACGAGGCTGAGGTTAAAA-3’ |
|  | C&R_IRF4-P4R | 5’-CAAGCACAGTCCCCAAAGTT-3’ |
| IRF4-P5 | C&R_IRF4-P5F | 5’-TCGTCGGTTTCATTCACCCA-3’ |
|  | C&R_IRF4-P5R | 5’-GGCAAAGCGGAGTCTTGTCT-3’ |
| Smu_1 | C&R_Smu_1F | 5’-CCACCTGGGTAATTTGCATTTC-3’ |
|  | C&R _Smu_1R | 5’-GGGAAACTAGAACTACTCAAGCTAA-3’ |
| Smu_3 | C&R _Smu_3F | 5’-TAGTAAGCGAGGCTCTAAAAAGCAC-3’ |
|  | C&R _Smu_3R | 5’-ACTCAGAGAAGCCCACCCAT-3’ |
| CpG methylation analysis | | |
| *Irf4*-MeP2 | MeP2F | 5’-TGGTTGTTTTTGTTTTTAGTTTGTG-3’ |
|  | MeP2R | 5’-CCCTCTTCTTCCTTTAAAATTTCAT-3’ |
| *Irf4*-MeP3 | MeP3F | 5’-GGGTTTTGATAATGGAAAATTAATT-3’ |
|  | MeP3R | 5’-CACCACCACAAATCAACTTAAAAC-3’ |
| *Irf4*-MeP4 | MeP4F | 5’-TTTATTTGGGGTATTGGTATTTTAT-3’ |
|  | MeP4R | 5’-ATCCCCAAAATTTCAATCATAAC-3’ |
| *Irf4*-MeP5 | MeP5F | 5’-GTTATGATTGAAATTTTGGGGATT-3’ |
|  | MeP5R | 5’-TCTCACAACATATAACAAAAAAAATAAACT-3’ |
| *Irf4*-MeP6 | MeP6F | 5’-ATAGGAGGAGTTGAAGAAAGTTAGG-3’ |
|  | MeP6R | 5’-AACAAAAAATCCCAAATTCAAATAATA-3’ |
