# Supplementary figures and images for: AID and TET2 cooperate to demethylate Irf4 for plasma cell fate in germinal center B cells
Source: J Exp Med. 2026 Apr 27;223(6):e20260096. doi: 10.1084/jem.20260096 (PMC13116153; doi:10.1084/jem.20260096)

Figure 8E\_AID IB

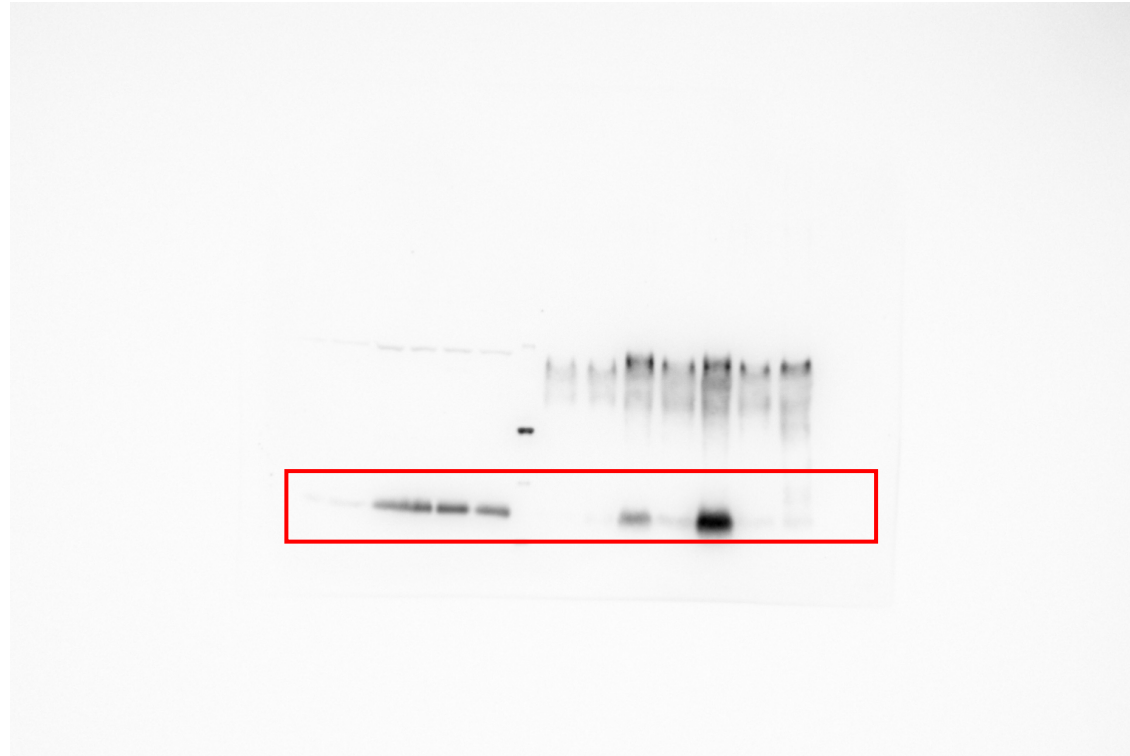

Figure 8E\_TET2 IB

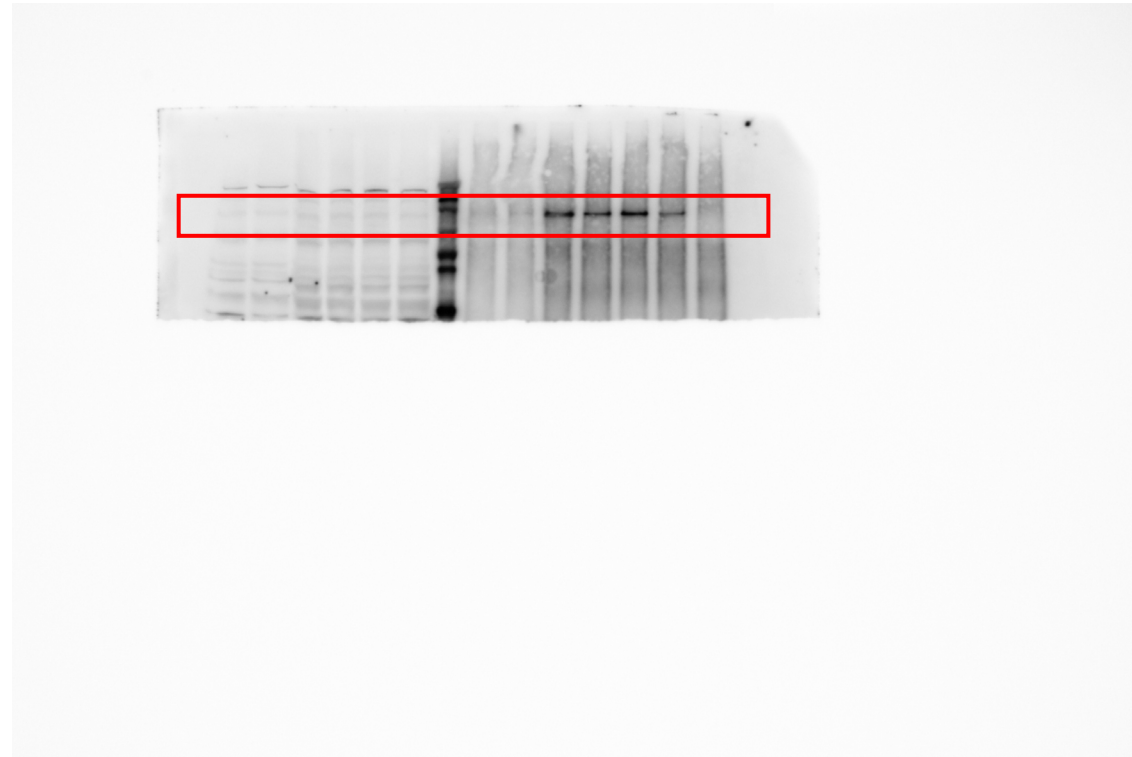

Supplement: SourceData F8 — is the source file for Fig. 8. [file jem_20260096_sourcedataf8.pdf]
