# Supplementary material for: AID and TET2 cooperate to demethylate Irf4 for plasma cell fate in germinal center B cells
Source: J Exp Med. 2026 Apr 27;223(6):e20260096. doi: 10.1084/jem.20260096 (PMC13116153; doi:10.1084/jem.20260096)

Supplemental Figure 4A_TET2-Flag IB

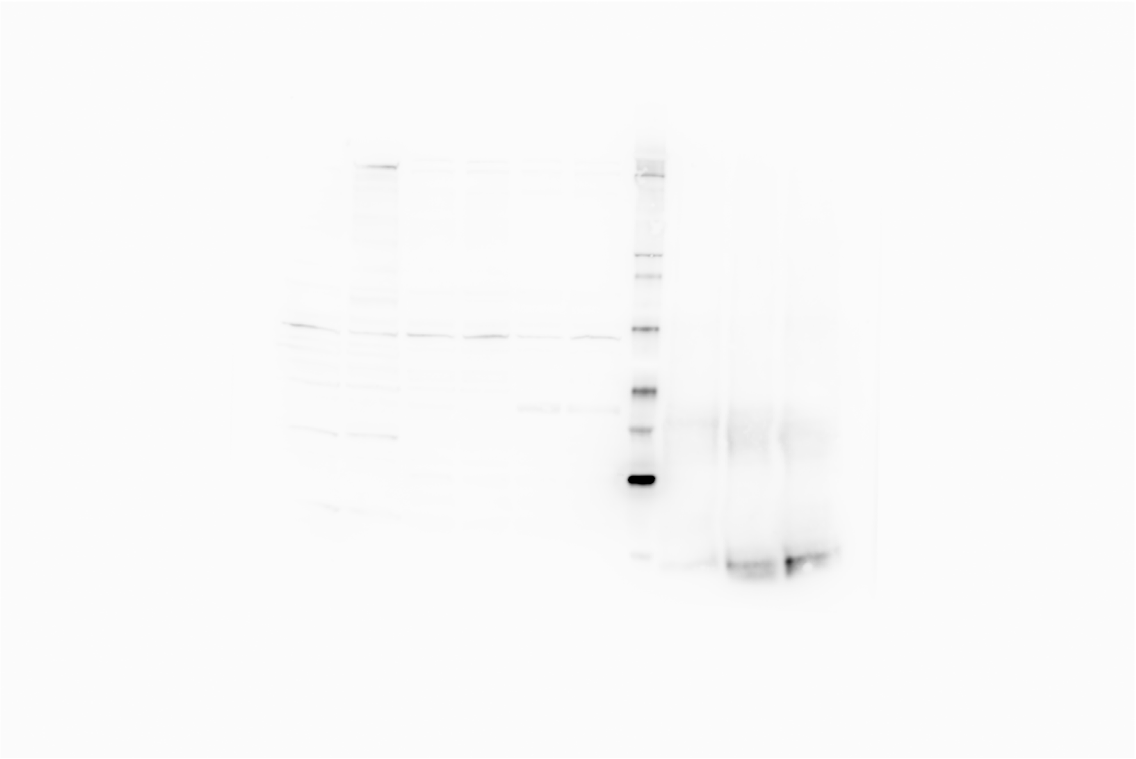

Supplemental Figure 4B_AID IB

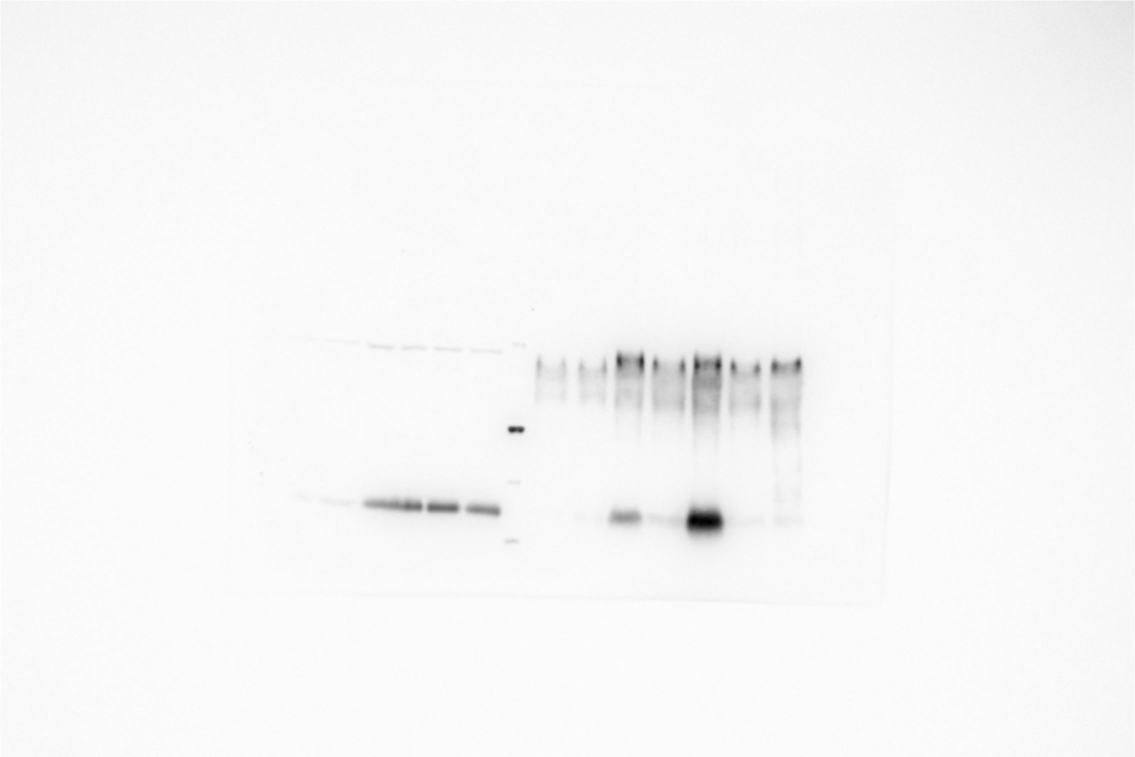

Supplemental Figure 4B_TET2 IB

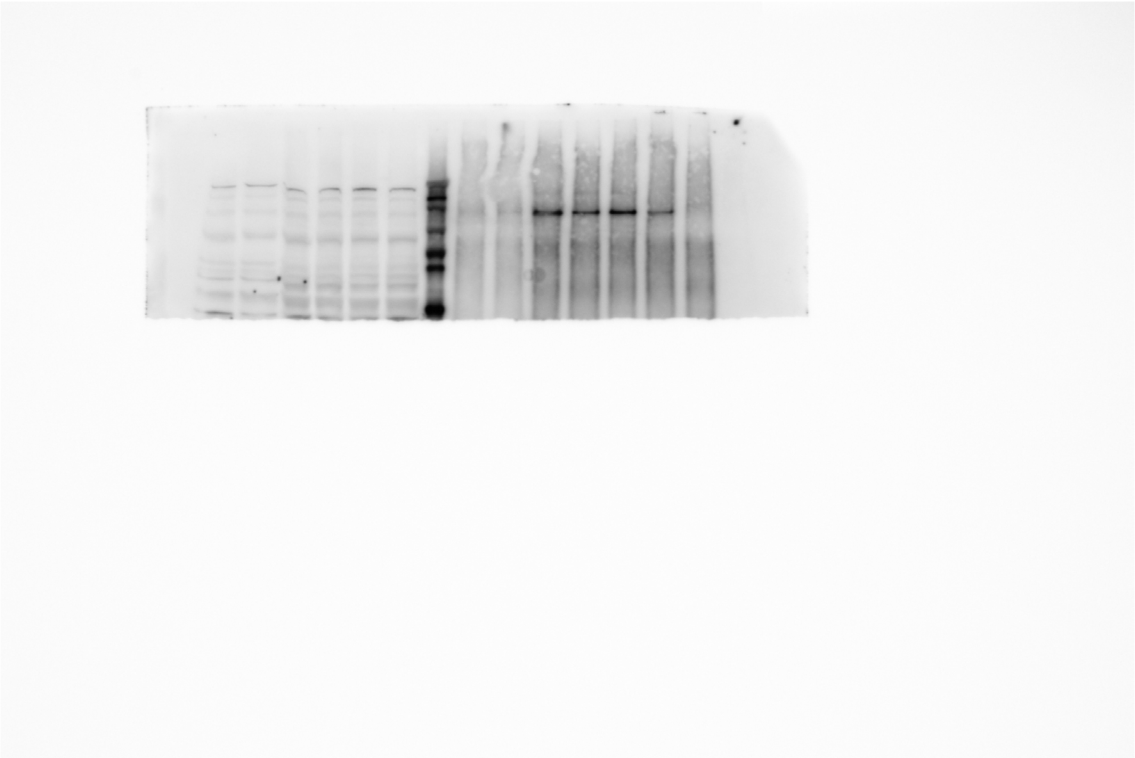

Supplemental Figure 4C_AID IB

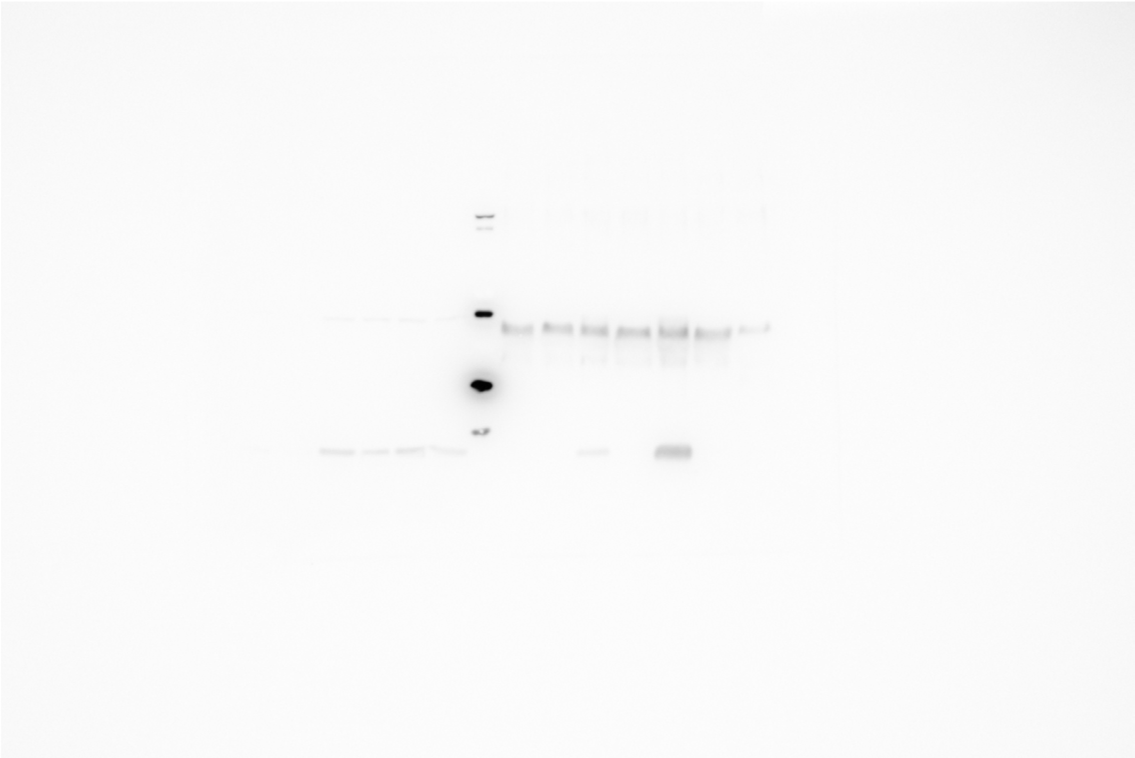

Supplemental Figure 4C_TET2 IB

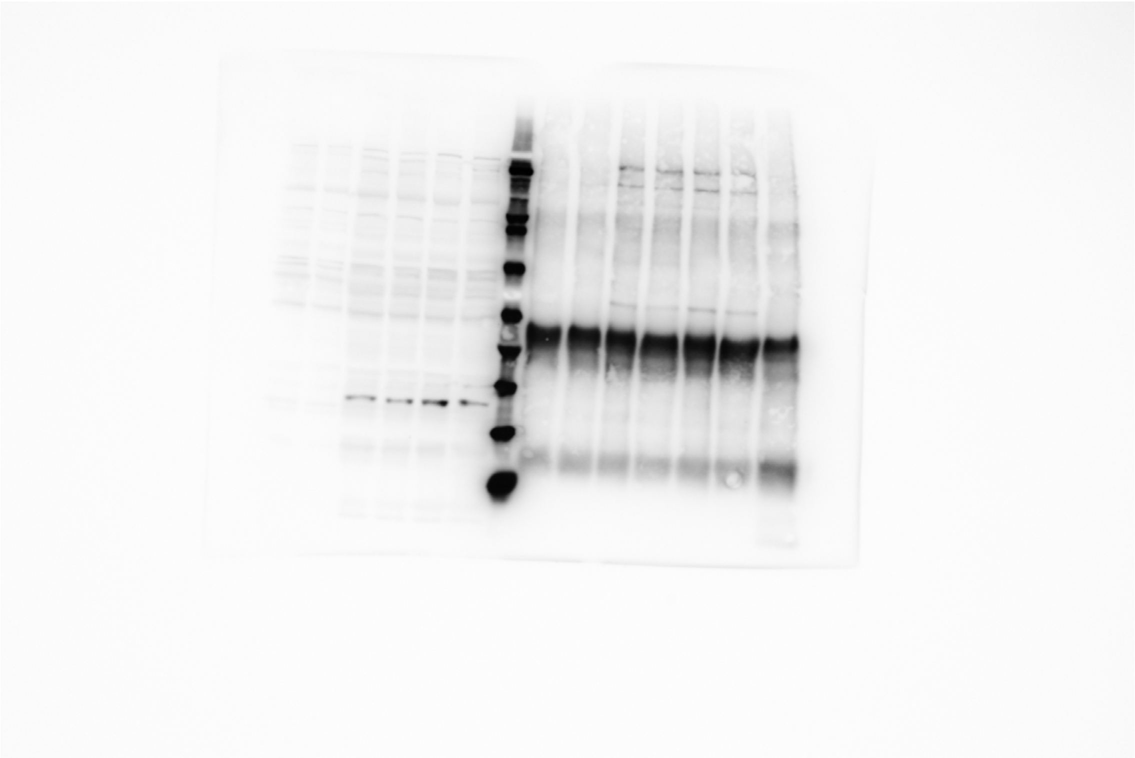

Supplemental Figure 4D_AID IB

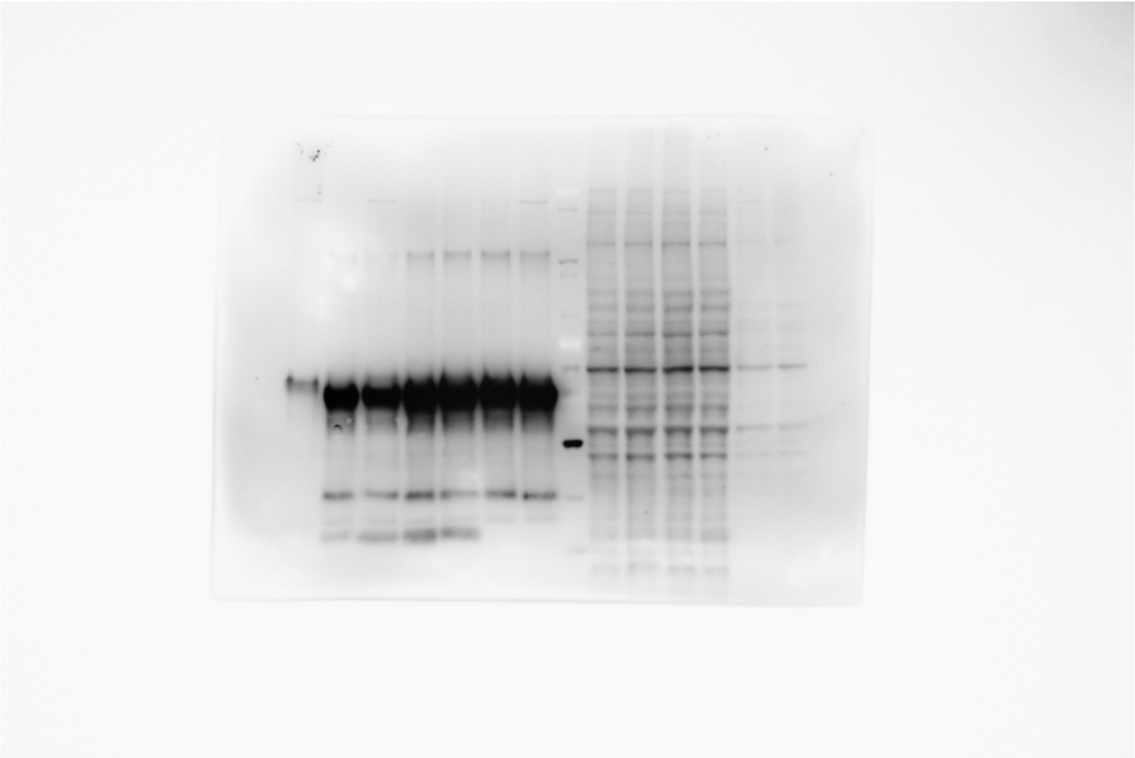

Supplemental Figure 4D_TET2 IB

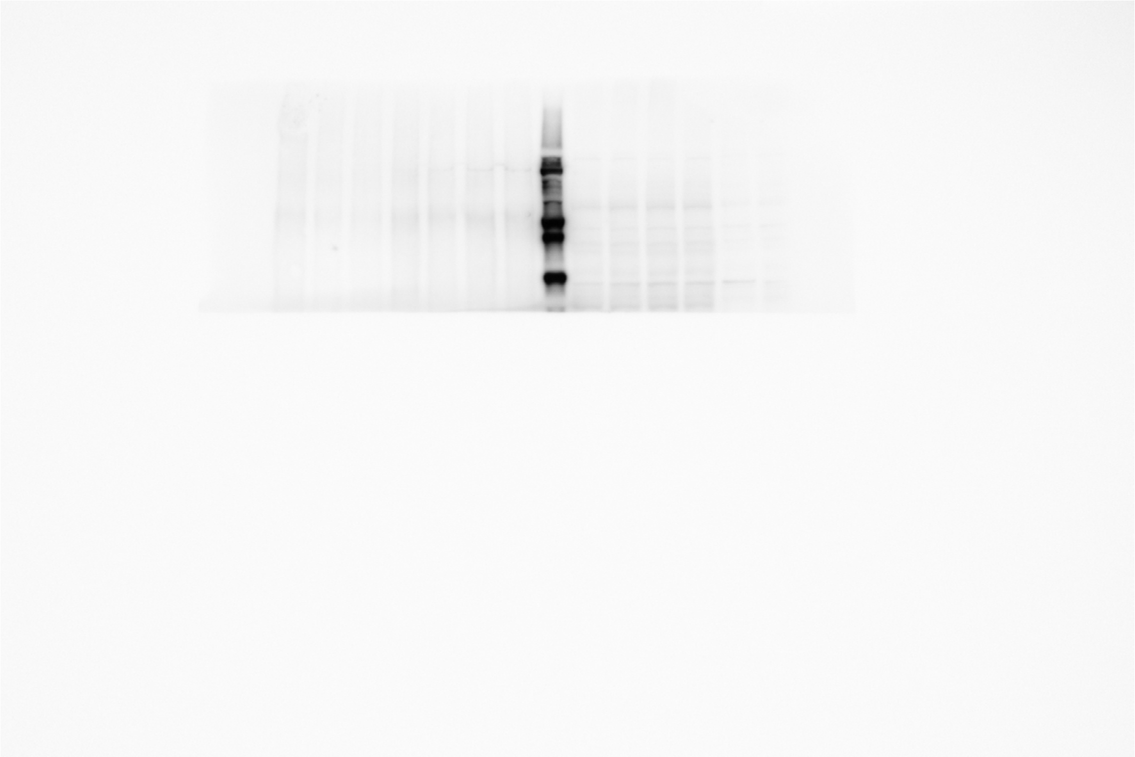

Supplemental Figure 4E_AID IB

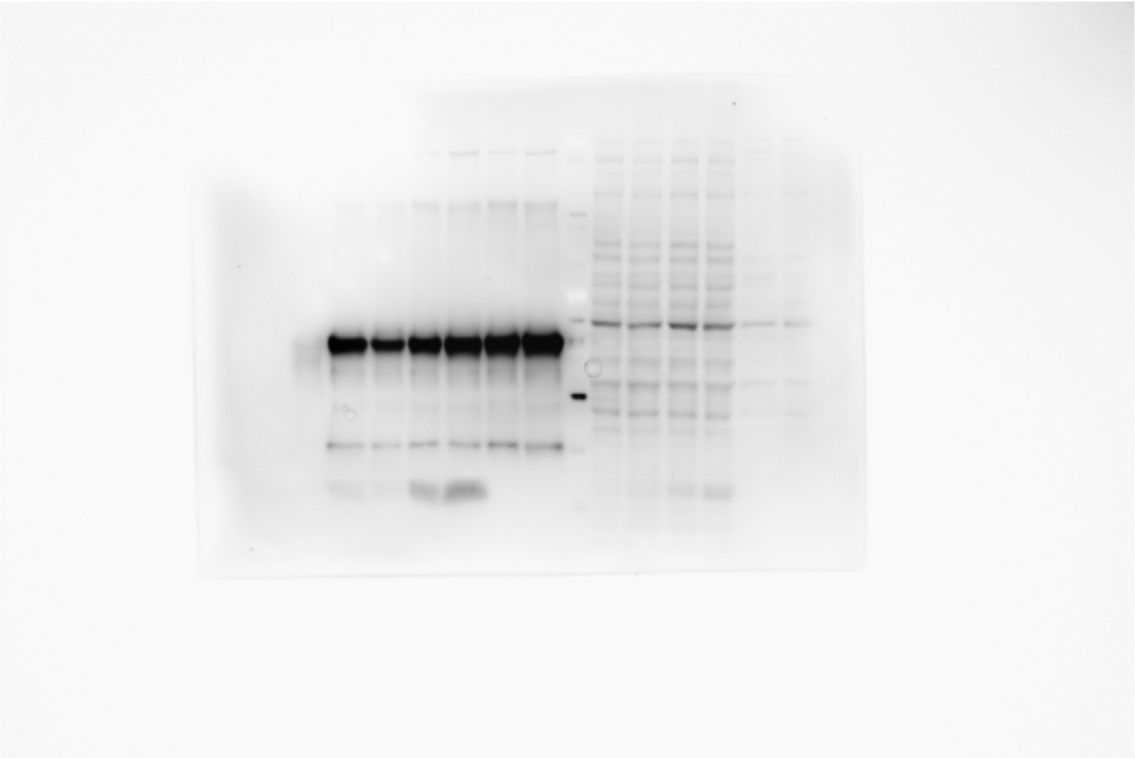

Supplemental Figure 4E_TET2 IB

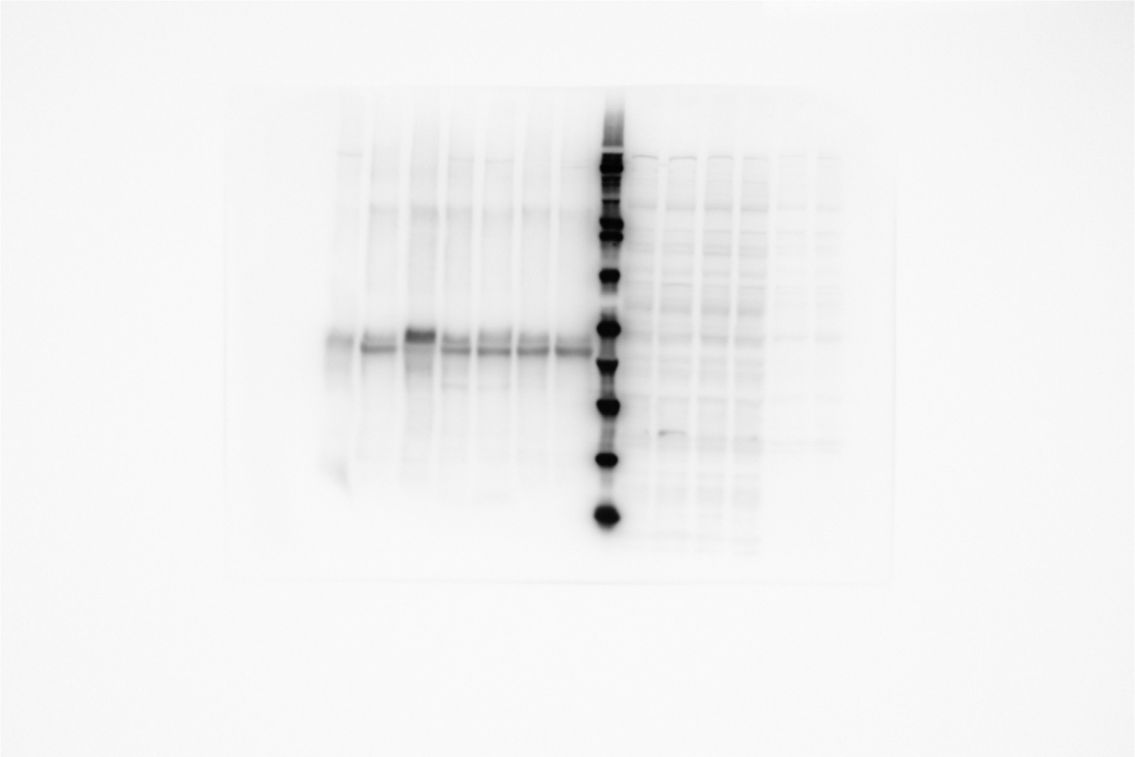

Supplemental Figure 4F_DNMT1&AID IB

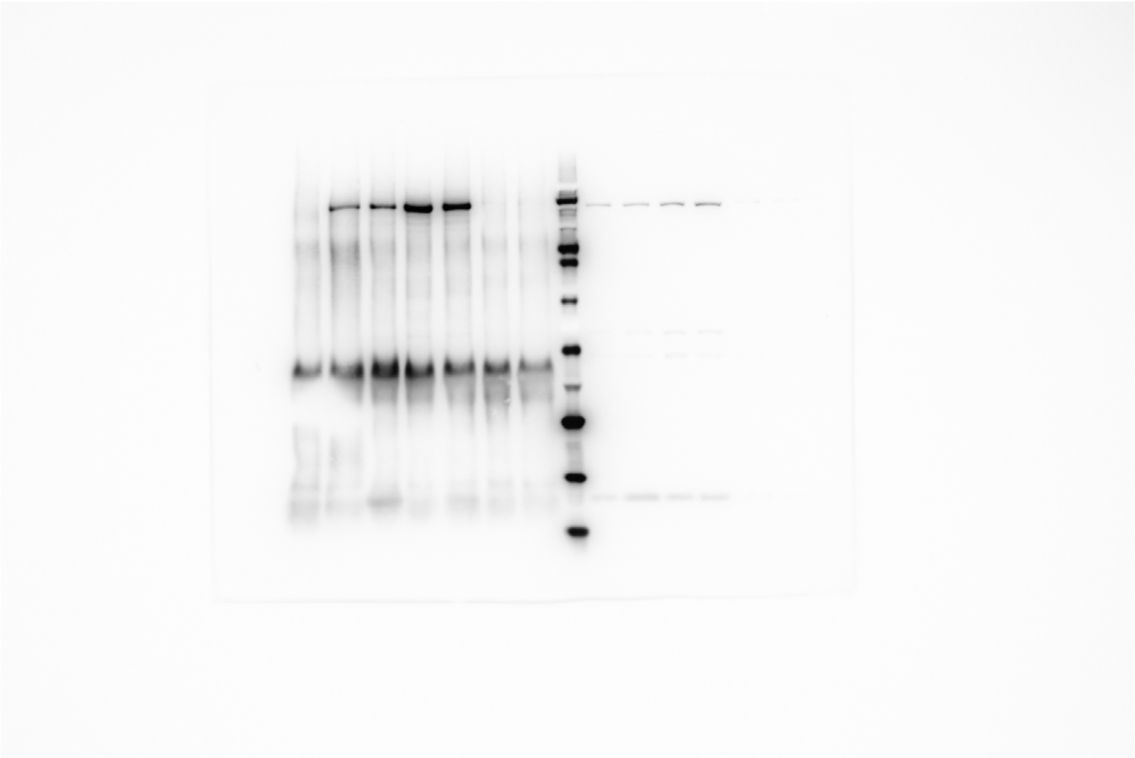

Supplement: SourceData FS4 — is the source file for Fig. S4. [file jem_20260096_sourcedatafs4.pdf]
